# Effects of Dietary Organic Acid Supplementation in Coccidiosis-Challenged Broilers: A Meta-Analysis

**DOI:** 10.3390/vetsci13090941

**Published:** 2026-09-10

**Authors:** Neslihan Ölmez, Roshan Riaz, Muhammad Waqas, Umair Ahsan, Ifrah Raza, Mükremin Ölmez, Ali R. Al Sulaiman, Ala E. Abudabos

**Affiliations:** 1 Department of Parasitology, Faculty of Veterinary Medicine, Kafkas University, Kars 36100, Türkiye; 2 Department of Animal Nutrition and Nutritional Diseases, Faculty of Veterinary Medicine, Kafkas University, Kars 36100, Türkiye; 3 Department of Poultry Production, Faculty of Animal Production and Technology, University of Veterinary and Animal Sciences, Lahore 54000, Pakistan; 4 Department of Plant and Animal Production, Burdur Vocational School of Food, Agriculture and Livestock, Burdur Mehmet Akif Ersoy University, Burdur 15030, Türkiye; uahsan@mehmetakif.edu.tr; 5 Department of Animal Nutrition and Nutritional Diseases, Faculty of Veterinary Medicine, Aydın Adnan Menderes University, Aydın 09016, Türkiye; raza_ifrah@hotmail.com; 6 Environmental Protection Technologies Institute, Sustainability and Environment Sector, King Abdulaziz City for Science and Technology, P.O. Box 6086, Riyadh 11442, Saudi Arabia; arsuliman@kacst.gov.sa; 7 Department of Food and Animal Sciences, College of Agriculture, Tennessee State University, Nashville, TN 37209, USA

**Keywords:** organic acids, coccidiosis, broiler chickens, *Eimeria* infection, lesion score, oocyst count (OPG), feed conversion ratio

## Abstract

This meta-analysis aimed to systematically assess the effects of organic acid supplementation on growth performance and disease-related parameters in broiler chickens challenged with coccidiosis. Following PRISMA guidelines, a systematic literature search was conducted across PubMed, Scopus, and Web of Science, yielding 11 controlled experimental studies for quantitative analysis. The outcomes measured were BWG, feed conversion ratio (FCR), lesion score, and oocyst count (OPG). No overall significant effect of organic acid supplementation was detected on body weight gain (BWG) (MD = 49.88 g; 95% CI: −156.94 to 256.70; *p* = 0.64) and FCR (MD = −0.12 g/g; 95% CI: −0.25 to 0.01; *p* = 0.08), with high heterogeneity across investigations (I^2^ = 100%) as identified through random-effects meta-analysis. Organic acid supplementation was associated with lower lesion scores (MD = −1.08 score units; 95% CI: −1.39 to −0.77; *p* < 0.00001; I^2^ = 57%) and reduced oocyst shedding (SMD = −1.25; 95% CI: −2.25 to −0.25; *p* = 0.01; I^2^ = 93%). Exploratory subgroup analyses suggested possible differences according to study duration, organic acid type, *Eimeria* species, and measurement time point. However, several subgroup estimates were based on a small number of studies and should therefore be interpreted cautiously rather than as confirmatory evidence of differential efficacy. Leave-one-out sensitivity analyses showed stable conclusions for BWG, FCR, and lesion score, whereas the statistical significance of the OPG effect was sensitive to omission of some studies. Exploratory assessments suggested possible small-study effects for BWG and OPG, but these analyses were underpowered because fewer than 10 studies contributed to each outcome. Using the GRADE framework, the certainty of evidence was rated as very low for BWG, FCR, lesion score, and OPG because of risk-of-bias concerns, inconsistency, and imprecision. Overall, very low-certainty evidence suggests that organic acids may reduce intestinal lesion severity and oocyst shedding, whereas their effects on growth performance remain uncertain. Further well-designed and standardized studies are required to clarify treatment effects and optimal supplementation strategies.

## Simple Summary

Coccidiosis is a common intestinal disease in broiler chickens that can damage the gut, reduce growth, and lower feed efficiency. Organic acids are feed additives that may help protect intestinal health and support disease control. This study combined results from 11 controlled experiments to evaluate whether adding organic acids to broiler diets improves growth and reduces the effects of coccidiosis. Overall, organic acid supplementation did not clearly improve body weight gain or feed efficiency. However, it was associated with less intestinal damage and lower shedding of the parasite in droppings. The effects varied considerably among studies because of differences in the type of organic acid, infection conditions, and experimental methods. The overall certainty of the available evidence was very low, so the findings should be interpreted cautiously. Organic acids may have value as complementary feed-based tools for supporting intestinal health and coccidiosis control, but further well-designed and standardized studies are needed before firm recommendations can be made.

## 1. Introduction

Coccidiosis is a protozoan disease caused by parasites of the genus *Eimeria* and remains one of the most economically important diseases in the global poultry industry. The early stages of the disease primarily target intestinal epithelial cells, leading to tissue destruction, impaired nutrient absorption, reduced feed production efficiency, and death when severe [1]. Such pathological effects result in substantial economic losses globally, reaching billions of dollars per year. Consistent experimental and meta-analytical evidence indicates that *Eimeria* infection negatively affects growth performance by reducing feed intake, body weight gain, and feed efficiency [2]. These consequences are mainly due to the destruction of the epithelial wall, impairment of intestinal barrier function, and dysbiosis, leading to nutrient leakage and malabsorption [3,4]. Taken together, these data point to the pressing need for control strategies that will reduce the impact of coccidiosis on broilers.

Anticoccidial drugs and antibiotic growth promoters have traditionally been used to control coccidiosis. Despite their long-term use and success, chemotherapeutic agents have posed significant challenges, including the emergence of *Eimeria* strains resistant to commonly used drugs and concerns about antimicrobial residues in poultry meat and eggs [5]. Surveillance conducted in various parts of the world has shown widespread and dynamic resistance towards commonly used anticoccidials among different *Eimeria* species. At the same time, public health concerns have been raised about residues in edible tissues and the development of antimicrobial resistance from antibiotic growth promoters [6]. These limitations have driven the search for safer, more sustainable alternatives to poultry production. This, together with the undesirable side effects of antibiotics, has recently increased research efforts regarding non-antibiotic-based strategies, such as vaccines, probiotics, phytogenics, and nutritional interventions like organic acids [7,8,9,10,11].

Among these alternatives, organic acids have been shown in several studies to serve as beneficial feed additives due to their combined effects on gut health and host resilience. Organic acids, including short-chain fatty acids such as butyrate, propionate, and formate, can lower pH in the gastrointestinal tract, inhibiting pathogenic microorganisms while promoting beneficial microbiota. They also enhance the digestibility of nutrients, intestinal morphology, and barrier function by modulating tight-junction protein expression and mucin production [12,13]. Empirical studies have indicated that the application of organic acids can inhibit pathogenic bacteria and stimulate beneficial microorganisms, in addition to improving growth performance and survival rate in broilers [14]. Moreover, organic acids have been found to reduce growth depression and enhance intestinal health indices, further supporting their application as substitutes for classical antimicrobials under disease-challenge conditions [11,15,16].

However, the effectiveness of organic acids, especially under coccidial challenge conditions, is inconsistent across studies. Other studies have reported improved growth performance without concomitant reductions in lesion scores or oocyst shedding [17]. In contrast, others have demonstrated reductions in both lesion severity and parasite load, accompanied by enhanced immune responses [15]. In addition, inconsistencies in outcomes, such as feed conversion ratio and ideal dosing, have been reported extensively [18]. Such discrepancies are influenced by several factors, including the type and formulation of organic acids, levels of addition, diet composition, and experimental conditions. Notably, the mechanisms underlying such effects remain to be fully clarified, with existing reviews concentrating on general broiler performance rather than a disease-specific context [19]. Hence, a systematic synthesis of evidence specifically targeting coccidiosis-challenged broilers is necessary. In this context, the current meta-analysis aims to systematically and quantitatively assess the impact of organic acid supplementation on growth performance and disease-related effects in poultry production systems, providing robust, evidence-based conclusions on their role.

## 2. Methodology

### 2.1. Search Strategy

This systematic review and meta-analysis was performed in accordance with the Preferred Reporting Items for Systematic Reviews and Meta-Analyses guidelines. It followed the methodological recommendations of the Cochrane Handbook for Systematic Reviews of Interventions [20]. A comprehensive, reproducible search strategy was developed to identify relevant studies evaluating the effects of coccidia-affected broiler chickens supplemented with organic acids. A combination of controlled vocabulary terms, such as Medical Subject Headings, and free-text search parameters was included in the search strategy to maximize sensitivity and specificity. Boolean operators (“AND” and “OR”) and database-specific field tags were used to retrieve relevant literature. A descriptive search structure was developed based on four conceptual domains: broiler chickens and poultry (1), coccidiosis and *Eimeria infection* (2), organic acids and associated products [short-chain fatty acids or short-chain organic acids] (3), and production performance/health-related outcome indicators, specifically FCR, BWG, lesion score, and OPG. Electronic searches were conducted using three key databases: PubMed/MEDLINE, Scopus, and Web of Science (WOS). The specific search strategies for each database are provided in Supplementary Table S1.

The literature search was conducted on 22 March 2026. All retrieved records were exported into reference management software such as EndNote (version 21.5; Clarivate Analytics, Philadelphia, PA, USA), where duplicate entries were identified and removed prior to title and abstract screening [21].

### 2.2. Eligibility Criteria

The eligibility criteria were specified a priori based on the PICOS (population, intervention, comparator, outcomes, and study design) framework. The population comprised broiler chickens experimentally challenged with coccidiosis due to *Eimeria* spp. Eligible interventions included dietary supplementation with organic acids, either as individual compounds (butyric, formic, acetic, and propionic acids) or in combination formulations. The comparator for the quantitative synthesis was a coccidiosis-challenged group receiving no organic acid supplementation. Studies containing additional antibiotic or standard anticoccidial treatment arms remained eligible, but these treatment arms were not used as comparators in the pooled meta-analysis. The main outcomes of interest were feed conversion ratio (FCR), body weight gain (BWG), lesion scores, and oocyst count (OPG). Studies had to provide adequate quantitative data for at least one outcome, specifically mean values together with measures of variability (standard deviation, standard error, or mean error) and sample size (*n*) per intervention and control group. The lesion scoring systems, OPG measurement units (e.g., ×10^3^ or ×10^5^), and log transformation status were also noted. Eligible study designs included controlled experimental trials conducted under coccidial-challenge conditions, regardless of randomization status. Studies not published in English were excluded. Studies were excluded if they were not controlled experimental trials, used a non-coccidial challenge model, did not employ an appropriate comparator group, or failed to report extractable outcome data for intervention and control groups. In addition, non-primary research articles, such as reviews, conference abstracts, case reports, and in vitro studies, were excluded from this analysis.

### 2.3. Study Selection and Screening

**Figure 1:**
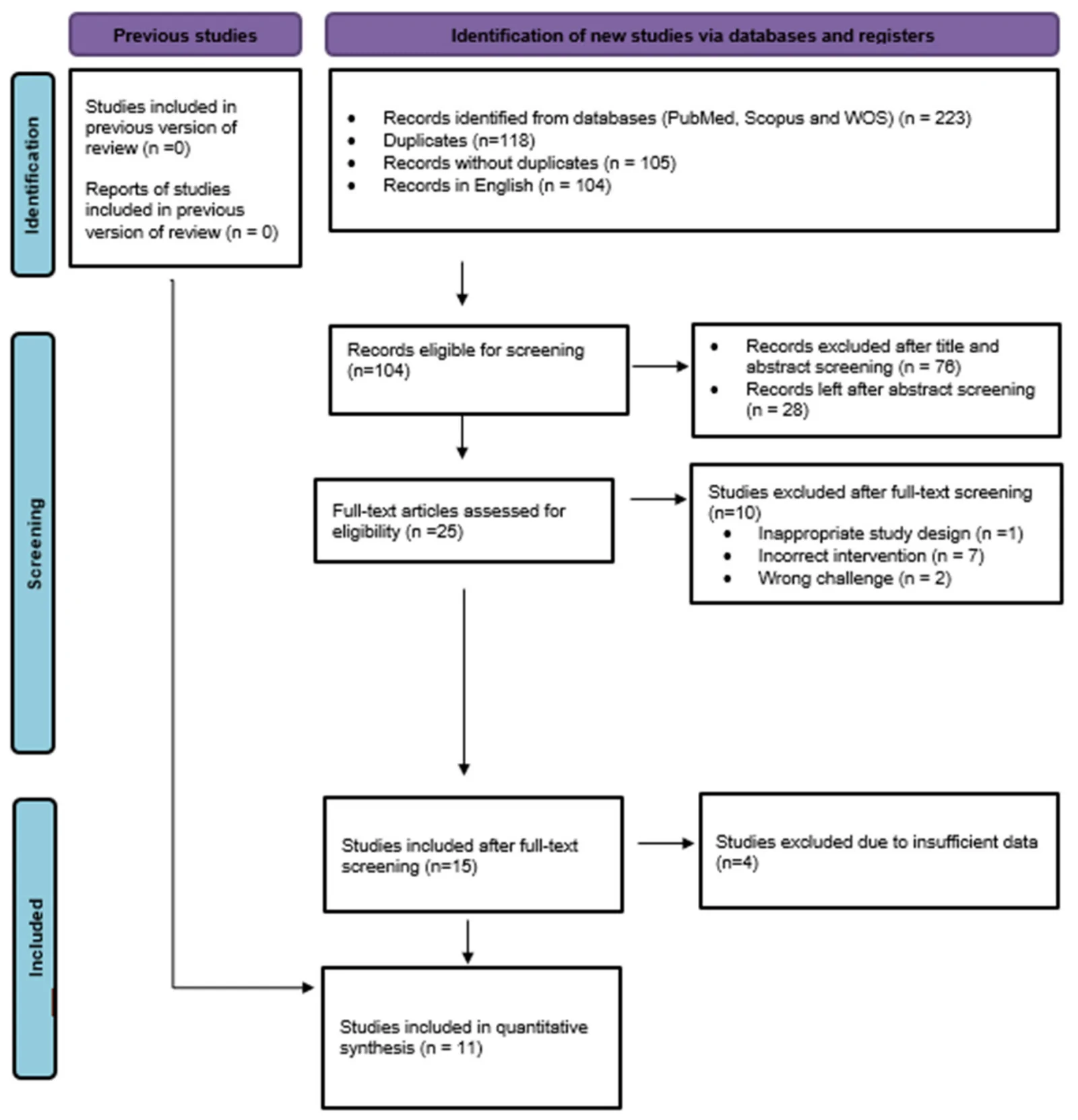
PRISMA 2020 flow diagram illustrating the selection process of studies investigating the effects of organic acid supplementation in broiler chickens challenged with coccidiosis [22,23,24,25,26,27,28,29,30,31,32].

Study screening was performed using a systematic two-stage process according to the PRISMA guidelines and recommendations in the Cochrane Handbook. The initial phase consisted of independent screening of titles and abstracts by two reviewers to identify potentially relevant studies based on explicitly reported information. Studies that appeared to fulfill the pre-established eligibility criteria or those in which there was insufficient data were retained for further analyses; clearly irrelevant studies were excluded. The screening process was conducted using the Rayyan systematic review tool, enabling efficient, blinded evaluation. Differences among reviewers were resolved through discussion and consensus. In the second stage, the full text of all potentially eligible studies was obtained and independently reviewed by the same two reviewers using predefined inclusion and exclusion criteria. Studies that fully met all criteria were included in the final analyses. Discrepancies were settled by discussion and consensus. Reasons for exclusion at the full-text stage were recorded to ensure transparency. The study selection procedures were conducted systematically to allow reproducibility and were summarized in accordance with the PRISMA flow diagram (Figure 1).

### 2.4. Data Extraction and Organization

Data extraction was performed in two consecutive phases, based on pre-established forms in accordance with internationally accepted methodological approaches to enable reproducibility. Two reviewers independently extracted data, and disagreements were resolved through discussion to reach consensus. During the first stage, study characteristics were extracted from all eligible studies included in the qualitative synthesis. The extracted variables included author and year, country, total sample size, broiler strain, age at challenge, *Eimeria* species, infection dose used, day of outcome measurement, organic acid type, dose and duration, comparator, and reported outcomes. Data were extracted from the full text as is, without further assumptions or changes. Standardized reporting was driven by universally applied decision rules that included detailed descriptions of the comparator groups, accurate naming of organic acid interventions, and precise reporting of dose, duration, and the time points of outcome measurement. Data were reported only for prespecified outcomes (FCR, BWG, lesion score, and OPG), and missing data were listed as “NR.” In the second stage, quantitative data for the meta-analysis were extracted in a structured manner, including study identifiers, experimental characteristics, intervention specifics, and outcome-specific statistics (mean, variability measures, and sample sizes for treatment and control groups). Data extraction was conducted separately for each outcome and time point. For performance outcomes (BWG and FCR), values for the overall study period were extracted. In contrast, for oocyst count (OPG), data were obtained at various time points, particularly 4 and 7 days post-infection (dpi), where possible. When multiple doses of the same organic acid intervention were reported within a study, the highest dose was selected according to a predefined extraction rule to retain a single intervention comparison and avoid incorporating multiple correlated effect estimates from the same study. This approach was used because the present meta-analysis focused on the overall effect of organic acid supplementation rather than dose–response relationships. When the results were reported as standard error (SE) or standard error of the mean (SEM), they were converted to standard deviation (SD) for consistency. Outcome measures were standardized using prespecified conversion rules where necessary. Body weight gain reported in grams and feed conversion ratio reported as g/g were retained in their original units because these units were consistent across studies. Where growth performance was reported as average daily gain, it was converted to total body weight gain for the corresponding experimental period by multiplying the reported daily mean by the number of days. The associated measures of variability were converted using the same multiplication factor. Lesion scores were retained on their reported scales because the included studies used comparable scoring directions, with higher scores indicating greater lesion severity. One lesion-score comparison was retained per study to avoid inclusion of multiple correlated estimates from the same experiment. The anatomical site of lesion assessment was recorded during extraction and varied across studies, including jejunal, cecal, and species-specific intestinal assessments; in one study, the precise anatomical region was not explicitly reported. Lesion outcomes were not selected according to the magnitude or statistical significance of the effect. Because only four studies contributed lesion-score data, subgroup analysis by anatomical region was not performed, and this variation was considered when interpreting the pooled estimate. OPG data were reported using different scales and transformations; therefore, these data were synthesized using the standardized mean difference rather than converted to a common absolute unit. Standardization was applied to outcome measurements only and not to organic acid inclusion levels, because the present meta-analysis was not designed as a dose–response analysis. Organic acid doses were extracted and reported as presented in the original studies.

### 2.5. Risk-of-Bias Assessment

The methodological quality of the studies was evaluated using the SYRCLE risk-of-bias tool for animal intervention studies [33]. The tool evaluates potential bias across the following domains: sequence generation, baseline characteristics, allocation concealment, randomization, blinding of caregivers/investigators, randomization of outcome assessment, blinding of outcome assessor, incomplete outcome data, selective outcome reporting, and other sources of bias. There were two processes for risk-of-bias assessment. In the first step, data relevant to the risk of bias were extracted from the full text of each included study using a standardized extraction form. We recorded only information that had been explicitly reported in the Methods, Results, tables, figures, or Supplementary Materials; any unreported information was noted as “Not reported.” In the second step, this information was used to make domain-level judgements of low, high, or unclear risk of bias, following SYRCLE’s guidance. For the study-level overall classification presented in the risk-of-bias figures, a conservative hierarchical rule was applied. Studies were classified as overall high risk of bias when at least one domain was rated high risk; as overall unclear risk when no domain was rated high risk but at least one domain was rated unclear; and as overall low risk only when all assessed domains were rated low risk. Two independent reviewers performed the extraction and judgement processes, and any disagreements were resolved through discussion and consensus.

### 2.6. Statistical Analysis

All statistical analyses were performed using R software (version 4.4.2; R Foundation for Statistical Computing, Vienna, Austria) with the metafor package (version 4.6-0) [34] and Review Manager (RevMan, version 5.4) [35]. Quantitative synthesis was conducted using appropriate meta-analytic approaches, and a random-effects model was applied to account for between-study variability [36]. Separate meta-analyses were performed for each of the four predefined outcomes: feed conversion ratio (FCR), body weight gain (BWG), lesion score, and oocyst count (OPG). Effect sizes for continuous outcomes were calculated as mean differences (MD) with corresponding 95% confidence intervals for body weight gain (BWG; g), feed conversion ratio (FCR; g/g), and lesion score (score units), as these outcomes were measured on comparable and consistent scales across studies. In contrast, oocyst count (OPG) was analyzed using standardized mean differences (SMD) due to variability in measurement units (e.g., ×10^3^, ×10^5^ oocysts/g) and the use of log-transformed data in some studies. Heterogeneity among studies was assessed using the I^2^ statistic and τ^2^ estimate, with statistical significance evaluated using Cochran’s Q-test. Subgroup analyses were conducted to explore potential sources of heterogeneity based on *Eimeria* species, type of organic acid, and time point of outcome measurement [37,38]. Sensitivity analysis was performed using a leave-one-out approach to assess the robustness of pooled estimates. Potential small-study effects were explored through visual inspection of funnel plots and Egger’s regression test, with the trim-and-fill method applied as an exploratory sensitivity analysis [39]. Because fewer than 10 studies were available for each outcome, these assessments were considered underpowered and were interpreted cautiously; no definitive conclusions regarding publication bias were drawn. Statistical significance was set at *p* < 0.05.

### 2.7. Certainty of Evidence Assessment

The certainty of evidence for each primary outcome (BWG, FCR, lesion score, and OPG) was assessed using the Grading of Recommendations Assessment, Development and Evaluation (GRADE) approach. Five domains were considered: risk of bias, inconsistency, indirectness, imprecision, and publication bias. Risk-of-bias judgments were informed by the SYRCLE assessment of the studies contributing to each outcome. Inconsistency was evaluated based on the magnitude of statistical heterogeneity, variability in effect estimates, and subgroup findings. Indirectness was assessed against the prespecified population, intervention, comparator, and outcomes of the review. Imprecision was evaluated according to the width of the 95% confidence interval, whether the interval crossed the null effect, and the amount of available information. Potential publication bias and small-study effects were considered using the exploratory funnel-plot and Egger’s test findings, recognizing their limited reliability when few studies were available. Evidence certainty was classified as high, moderate, low, or very low, with downgrading by one or two levels for serious or very serious concerns, respectively.

## 3. Results

### 3.1. Study Selection

The study selection process was conducted in accordance with PRISMA 2020 guidelines to ensure transparency and reproducibility. A total of 223 records were identified through database searching, including 55 from PubMed, 81 from Scopus, and 87 from Web of Science. After the removal of 118 duplicate records using reference management software (EndNote), 105 unique studies remained. Among these, one study published in Portuguese was excluded, resulting in 104 records eligible for title and abstract screening. During title and abstract screening using Rayyan, 76 records were excluded based on predefined eligibility criteria, leaving 28 studies for further consideration. Of these, 25 studies were deemed eligible for full-text screening. Following full-text evaluation, 10 studies were excluded with specific reasons, including inappropriate study design (n = 1) [40], incorrect intervention (n = 7) [41,42,43,44,45,46,47], and absence of a relevant coccidial challenge model (n = 2) [48,49]. An additional four studies were excluded due to insufficient statistical data required for meta-analysis [50,51,52,53]. Ultimately, 11 studies met all inclusion criteria and were included in the final quantitative synthesis. The complete study selection process is presented in the PRISMA flow diagram (Figure 1).

### 3.2. Study Characteristics

The characteristics of the studies included in the meta-analysis are summarized in Table 1, including study setting, broiler characteristics, coccidial challenge model, intervention details, and outcome measurements. The methodological and experimental conditions of the included studies are summarized in Table 1 to facilitate an understanding of the factors affecting the pooled estimates. There was heterogeneity among the included studies in terms of broiler strain, age at challenge, *Eimeria* species, doses of infection per animal, organic acid formulation used, time points for outcomes, and details on the intervention protocol. These variabilities demonstrate the heterogeneity of findings across studies, which was appropriately addressed using a random-effects model for the meta-analysis. Detailed study-level information is provided in Table 1 as well as in Supplementary Table S2 to ensure transparency and reproducibility of the analyses.

### 3.3. Risk-of-Bias Findings

**Figure 2:**
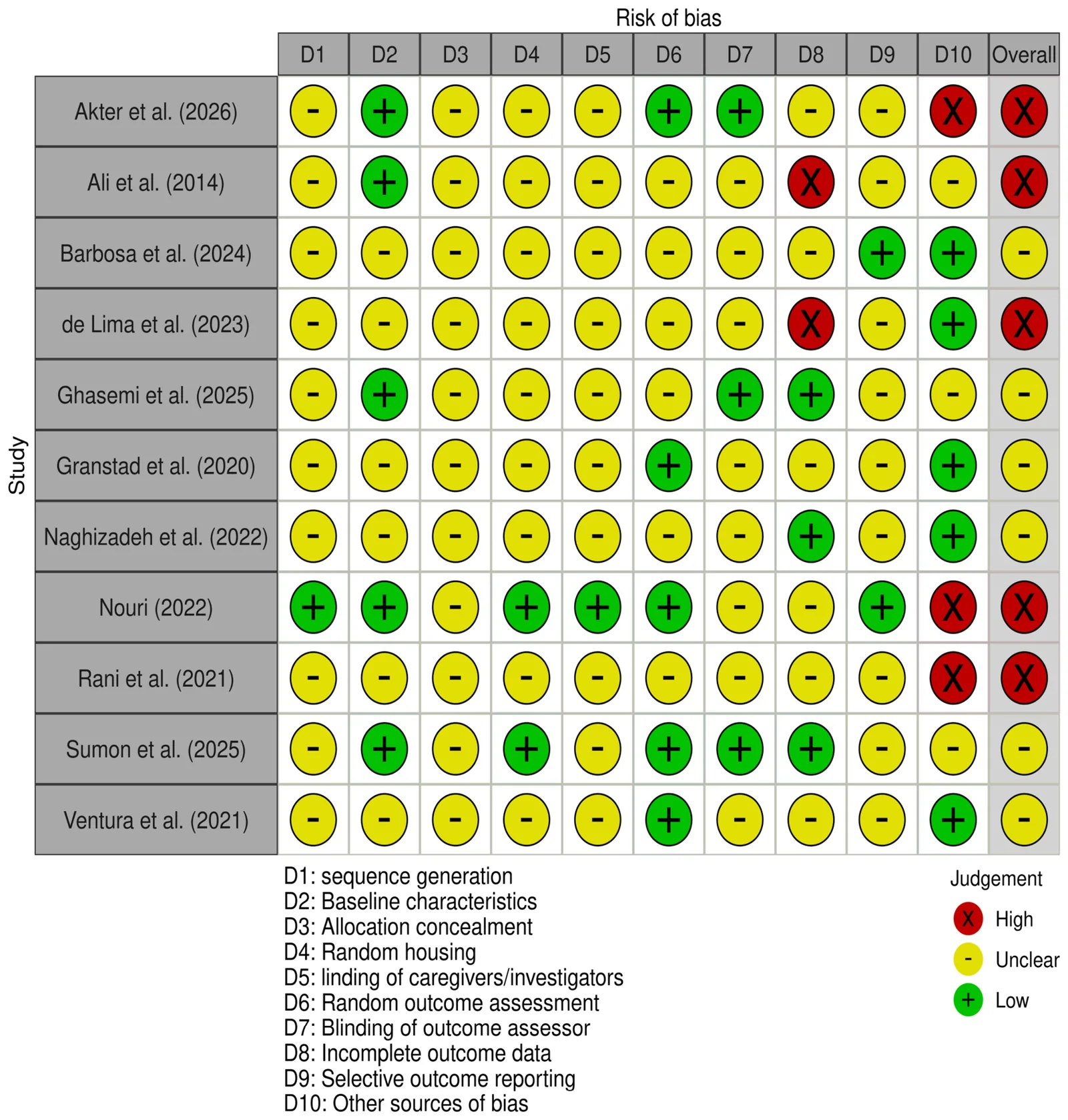
Traffic light plot of SYRCLE risk-of-bias assessment across included studies [22,23,24,25,26,27,28,29,30,31,32].

**Figure 3:**
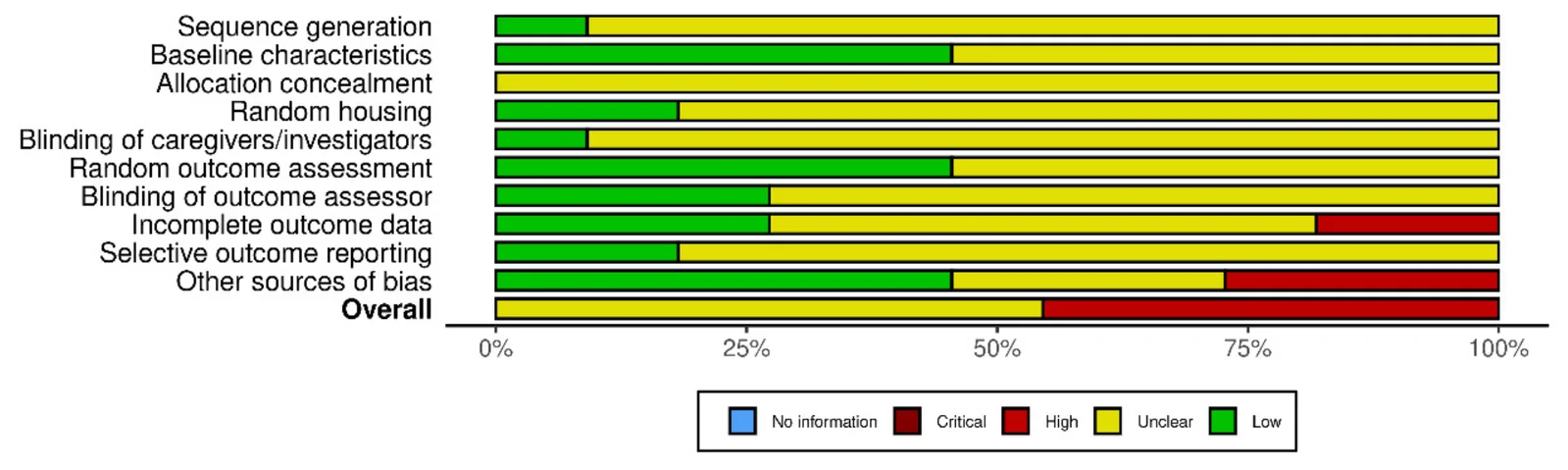
Summary plot of SYRCLE risk-of-bias assessment across all domains [22,23,24,25,26,27,28,29,30,31,32].

The SYRCLE Risk of Bias tool was used to assess risk of bias across the major methodological domains, including sequence generation, allocation concealment, randomization, blinding of caregivers/investigators and outcome assessors, random outcome assessment, incomplete outcome data or missing results, selective reporting, and other sources of bias. The assessment was conducted systematically according to predetermined criteria to ensure uniformity across studies. The study-level risk-of-bias assessments are shown in the traffic light plot (Figure 2), and an overall summary plot is provided for all domains (Figure 3).

### 3.4. Body Weight Gain (BWG)

**Figure 4:**
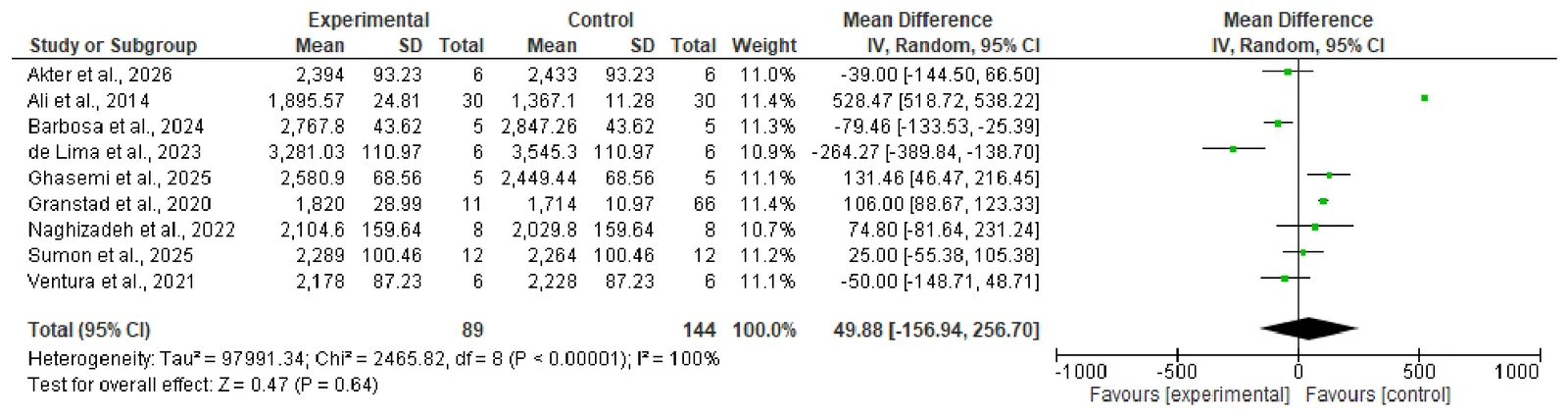
Random-effects meta-analysis (IV method) showing no significant effect of organic acids on body weight gain (BWG) [22,23,24,25,26,27,28,31,32].

The meta-analysis included 9 studies comprising 233 experimental units (89 in the treatment group and 144 in the control group). Using a random-effects model, the pooled mean difference (MD) was 49.88 g (95% CI: −156.94 to 256.70), indicating no statistically significant effect (Z = 0.47, *p* = 0.64). Substantial heterogeneity was observed among the included studies (τ^2^ = 97,991.34; χ^2^ = 2465.82, df = 8, *p* < 0.00001; I^2^ = 100%), as shown in Figure 4.

**Figure 5:**
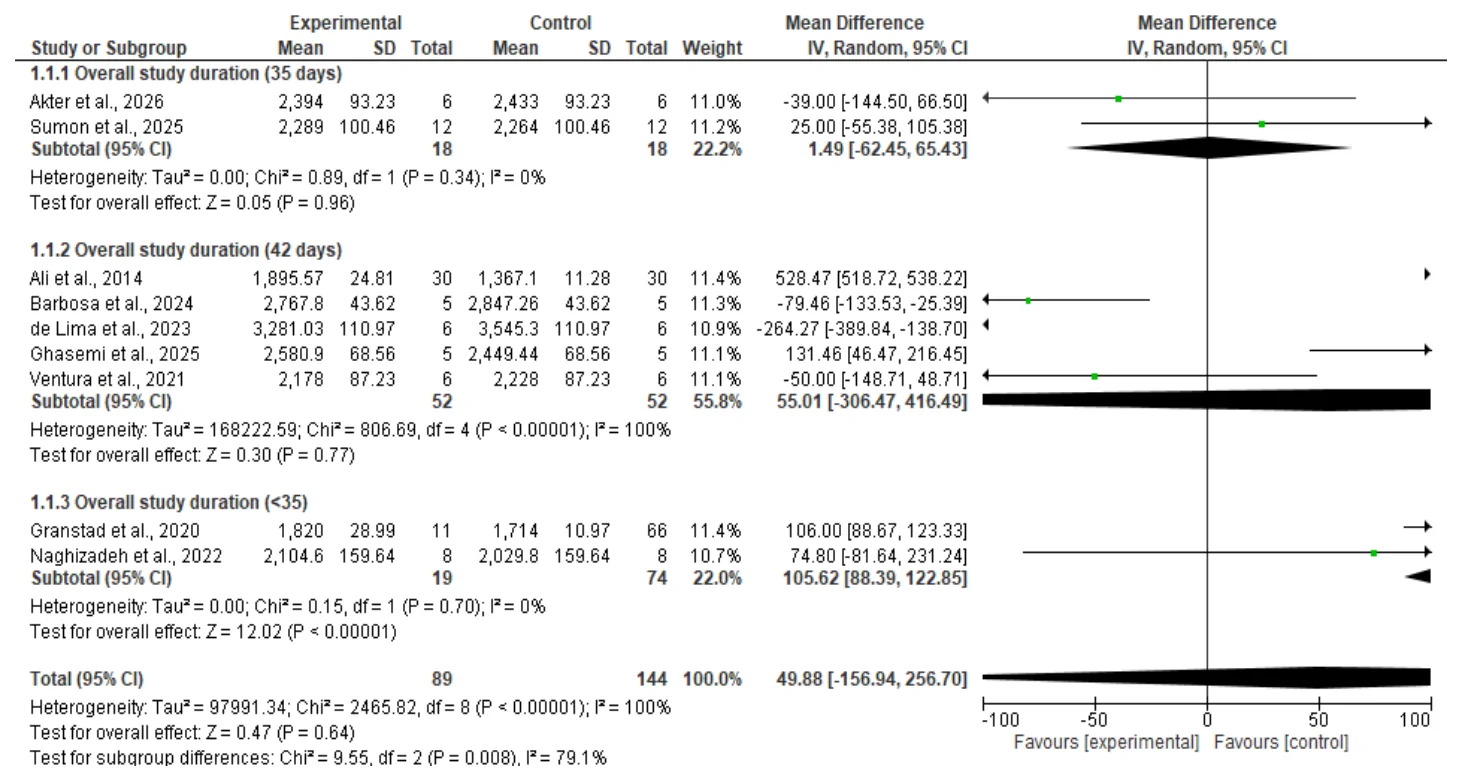
Subgroup analysis by study duration shows no significant effect on BWG at 35 and 42 days but a significant increase at <35 days, with substantial between-subgroup heterogeneity (*p* = 0.008) [22,23,24,25,26,27,28,31,32].

Subgroup analysis based on study duration showed that studies with a duration of <35 days demonstrated a significant effect (MD = 105.62 g, 95% CI: 88.39 to 122.85; Z = 12.02, *p* < 0.00001; I^2^ = 0%), whereas studies with durations of 35 days (MD = 1.49 g, 95% CI: −62.45 to 65.43; *p* = 0.96; I^2^ = 0%) and 42 days (MD = 55.01 g, 95% CI: −306.47 to 416.49; *p* = 0.77; I^2^ = 100%) showed no significant effects. A significant subgroup difference was detected (χ^2^ = 9.55, df = 2, *p* = 0.008; I^2^ = 79.1%), as shown in Figure 5.

**Figure 6:**
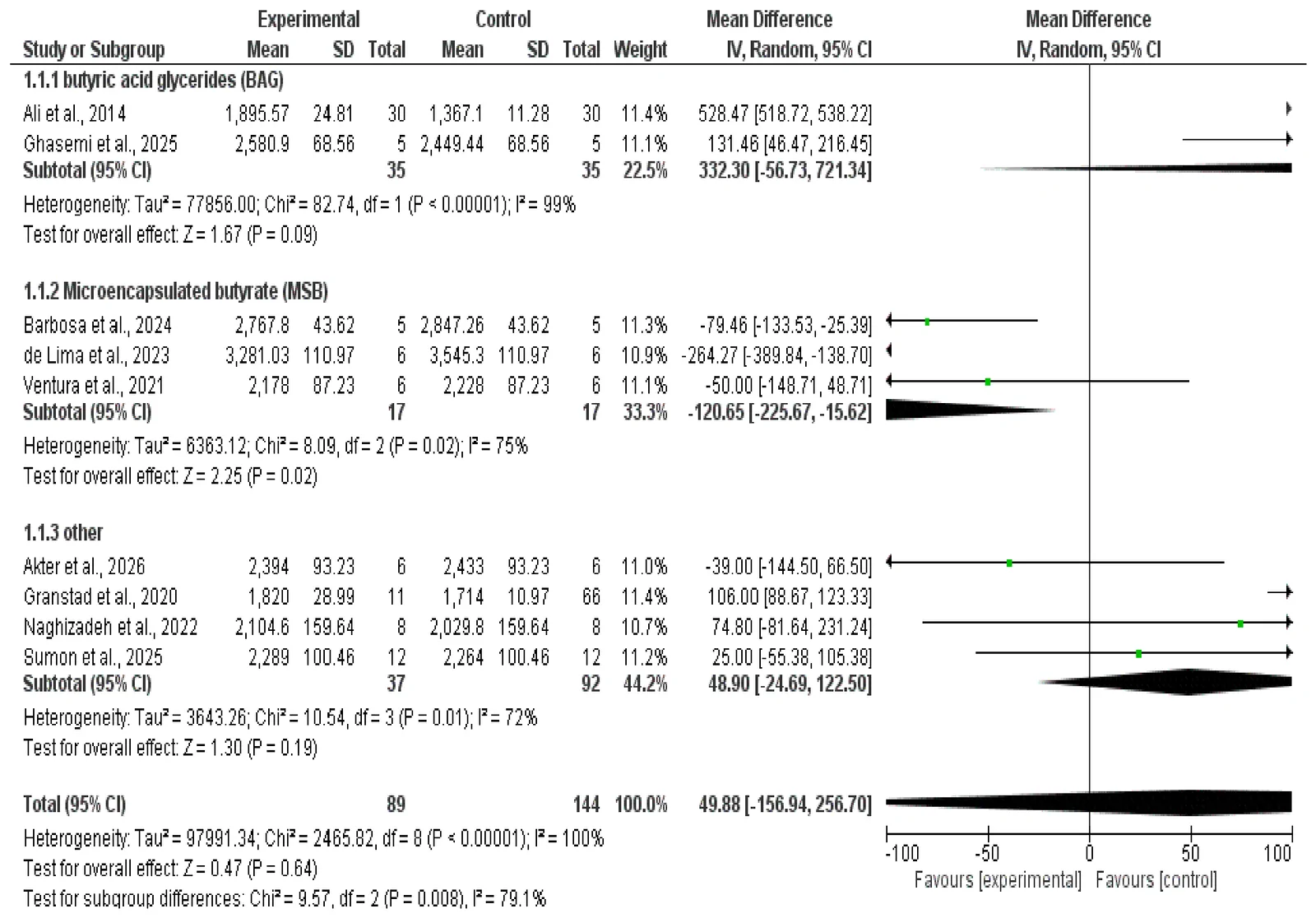
Subgroup analysis by organic acid type shows a significant reduction in BWG for microencapsulated butyrate, while other forms showed no significant effect, with substantial between-subgroup heterogeneity (*p* = 0.008) [22,23,24,25,26,27,28,31,32].

Subgroup analysis based on organic acid type indicated no significant effect for butyric acid glycerides (MD = 332.30 g, 95% CI: −56.73 to 721.34; *p* = 0.09; I^2^ = 99%) or other organic acids (MD = 48.90 g, 95% CI: −24.69 to 122.50; *p* = 0.19; I^2^ = 100%), whereas microencapsulated sodium butyrate showed a significant effect (MD = −120.65 g, 95% CI: −225.67 to −15.62; *p* = 0.02; I^2^ = 75%). The overall pooled estimate remained non-significant (MD = 48.88 g, 95% CI: −156.94 to 256.70; *p* = 0.64; I^2^ = 100%), with a significant subgroup difference (χ^2^ = 9.57, df = 2, *p* = 0.008; I^2^ = 79.1%), as shown in Figure 6.

**Figure 7:**
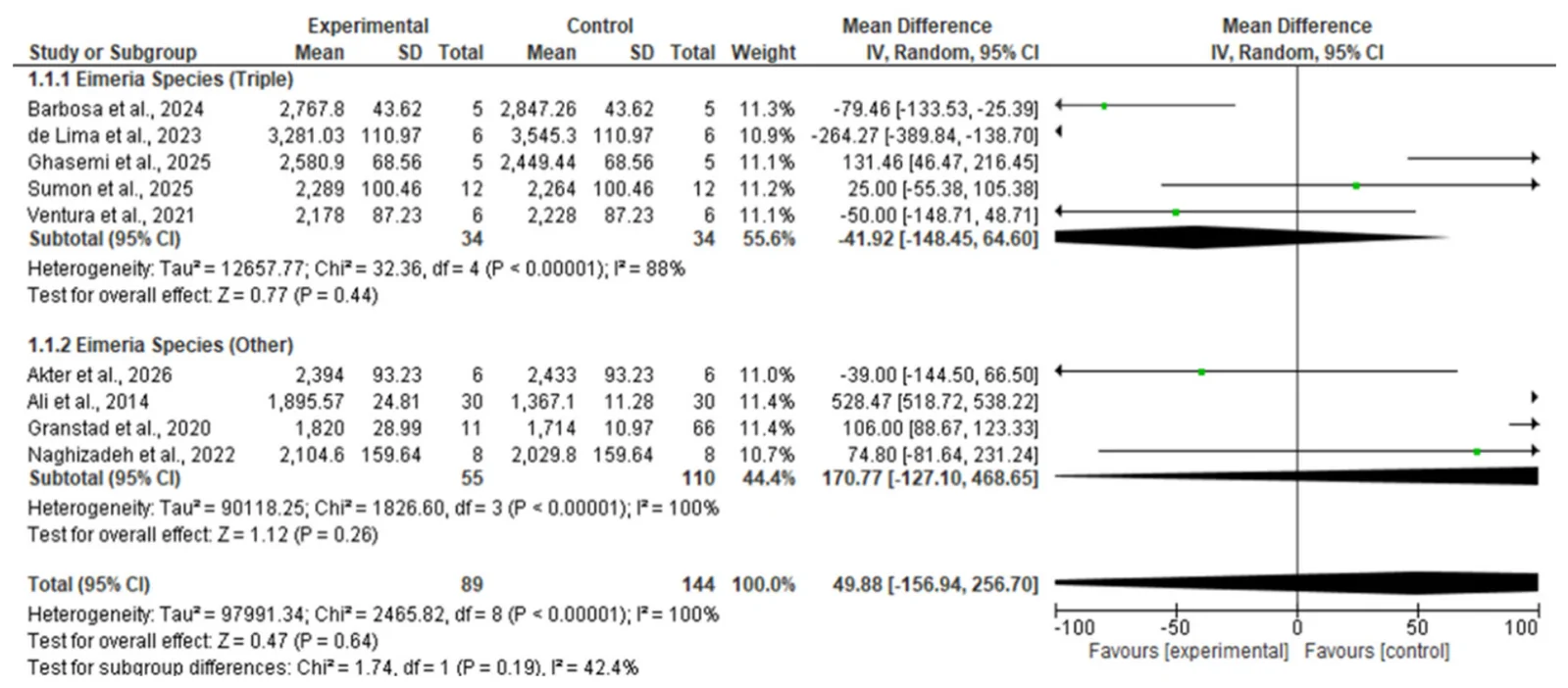
Subgroup analysis by *Eimeria* species shows no significant effect on BWG in either multiple-species or other-species infections, with high heterogeneity and no significant subgroup difference (*p* = 0.19) [22,23,24,25,26,27,28,31,32].

Subgroup analysis based on *Eimeria* species showed no significant effects for triple-species infection (MD = −41.92 g, 95% CI: −148.45 to 64.60; *p* = 0.44; I^2^ = 88%) or other species (MD = 170.77 g, 95% CI: −127.10 to 468.65; *p* = 0.26; I^2^ = 100%), with no significant subgroup difference (χ^2^ = 1.74, df = 1, *p* = 0.19; I^2^ = 42.4%), as shown in Figure 7.

**Figure 8:**
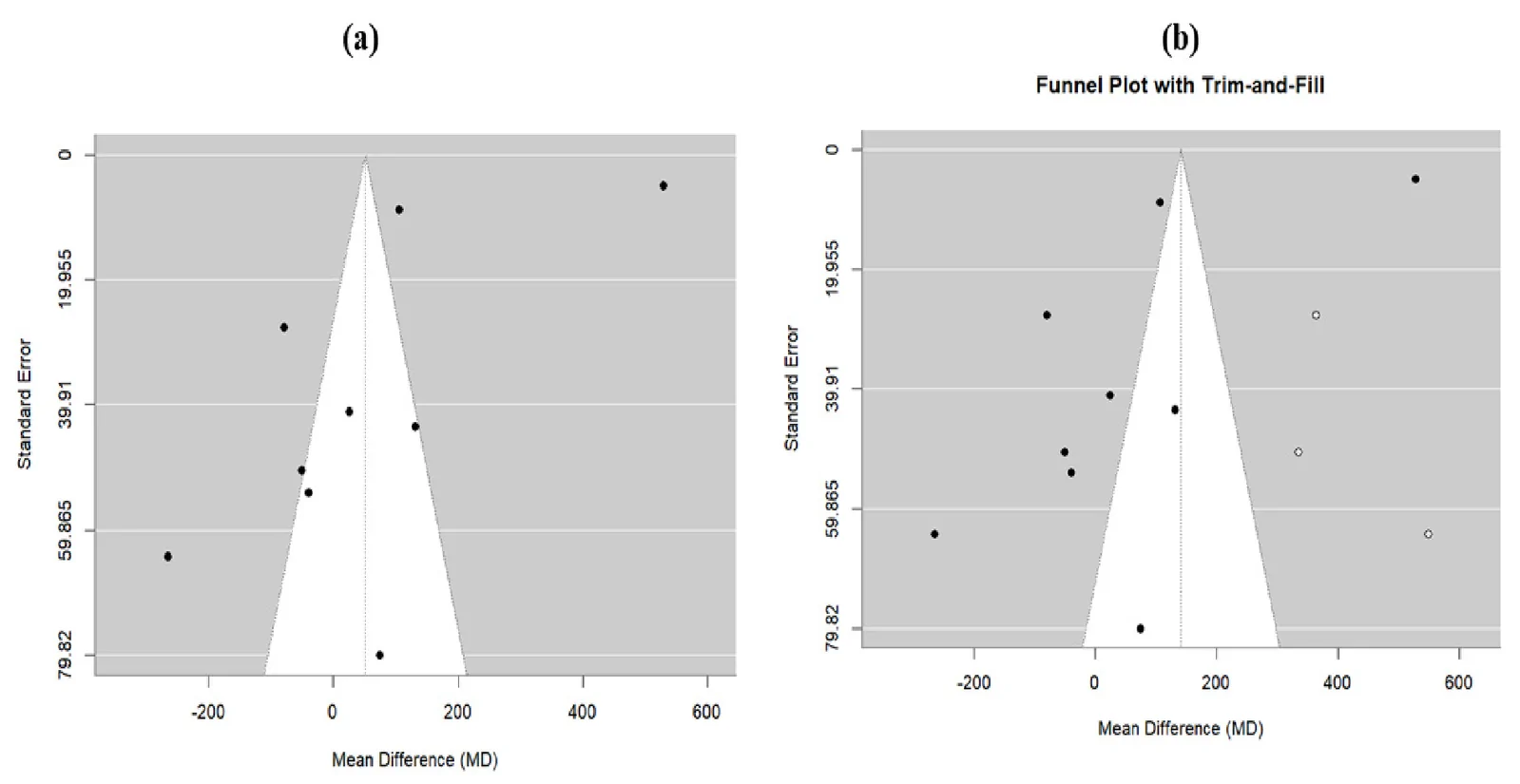
(**a**) Funnel plot suggests potential asymmetry, and (**b**) trim-and-fill analysis indicates possible missing studies.

Sensitivity analysis using a leave-one-out approach showed that the pooled estimates ranged from −8.36 g (95% CI: −93.57 to 76.84; *p* = 0.85) to 90.11 g (95% CI: −49.41 to 229.63; *p* = 0.21) and remained non-significant across all iterations, indicating that no single study disproportionately influenced the overall effect. Exploratory publication bias assessment showed asymmetry in the funnel plot, which was further supported by Egger’s regression test (z = −2.061, *p* = 0.039). The trim-and-fill method suggested potential missing studies on the right side of the funnel plot, with a bias-adjusted pooled mean difference of 276.06 g (95% CI: 32.24 to 519.87), as shown in Figure 8.

### 3.5. Feed Conversion Ratio (FCR)

**Figure 9:**
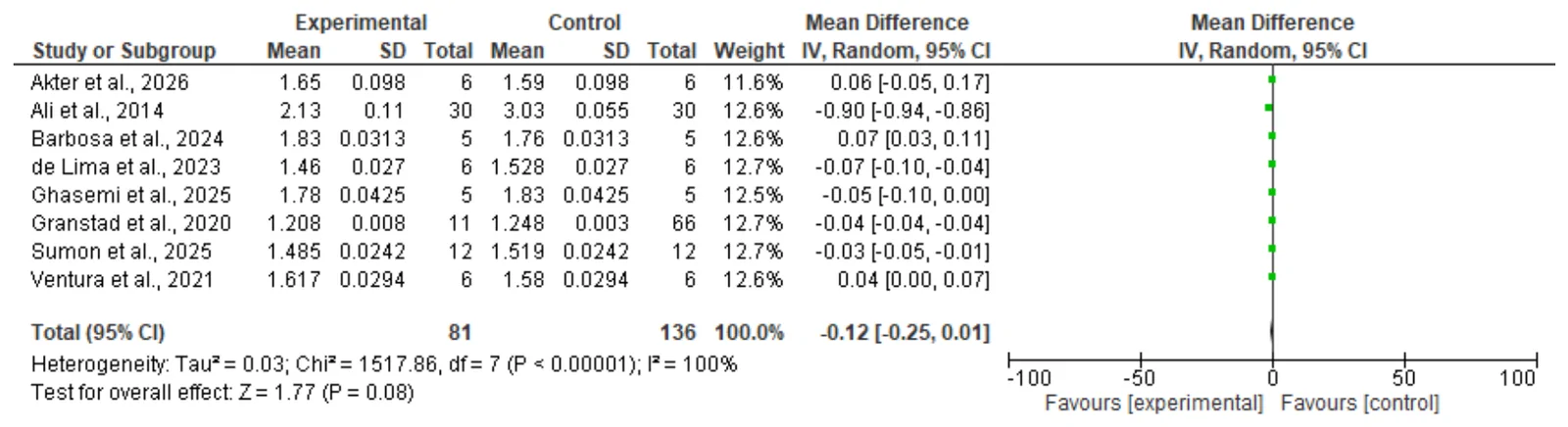
Forest plot showing the effect of organic acids on feed conversion ratio (FCR), indicating no statistically significant overall effect with substantial heterogeneity [22,23,24,25,26,27,31,32].

The meta-analysis included 8 studies comprising 217 experimental units (81 in the treatment group and 136 in the control group). Using a random-effects model, the pooled mean difference (MD) was −0.12 g/g (95% CI: −0.25 to 0.01), indicating no statistically significant overall effect (Z = 1.77, *p* = 0.08). Considerable heterogeneity was observed among studies (τ^2^ = 0.03; χ^2^ = 1517.86, df = 7, *p* < 0.00001; I^2^ = 100%), as shown in Figure 9.

**Figure 10:**
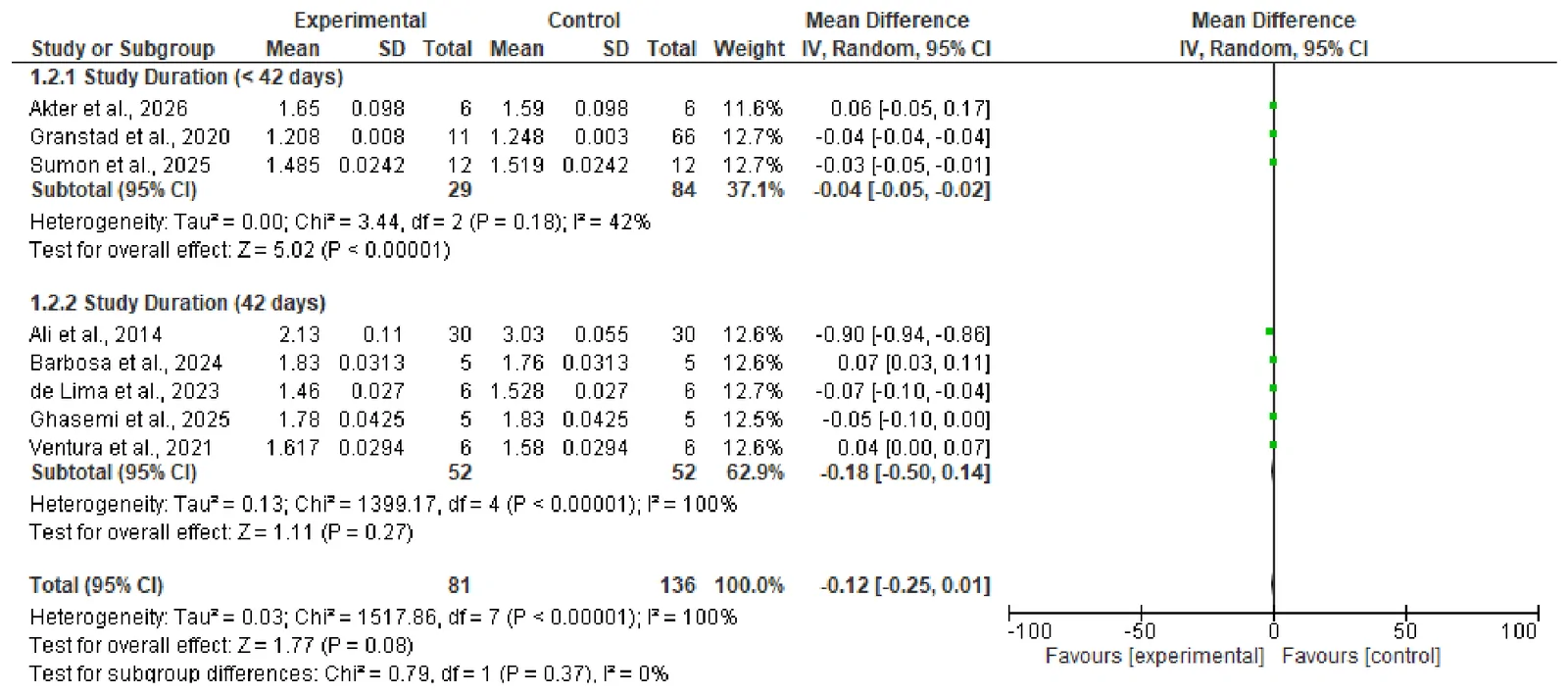
Subgroup forest plot by study duration for feed conversion ratio (FCR), showing a significant effect in studies < 42 days but not in 42-day studies, with no significant subgroup difference [22,23,24,25,26,27,31,32].

Subgroup analysis based on study duration showed a statistically significant effect in studies with duration <42 days (MD = −0.04 g/g, 95% CI: −0.05 to −0.02; Z = 5.02, *p* < 0.00001; I^2^ = 42%), whereas studies with 42 days duration showed no significant effect (MD = −0.18 g/g, 95% CI: −0.50 to 0.14; *p* = 0.27; I^2^ = 100%), with no significant subgroup difference (χ^2^ = 0.79, df = 1, *p* = 0.37; I^2^ = 0%), as shown in Figure 10.

**Figure 11:**
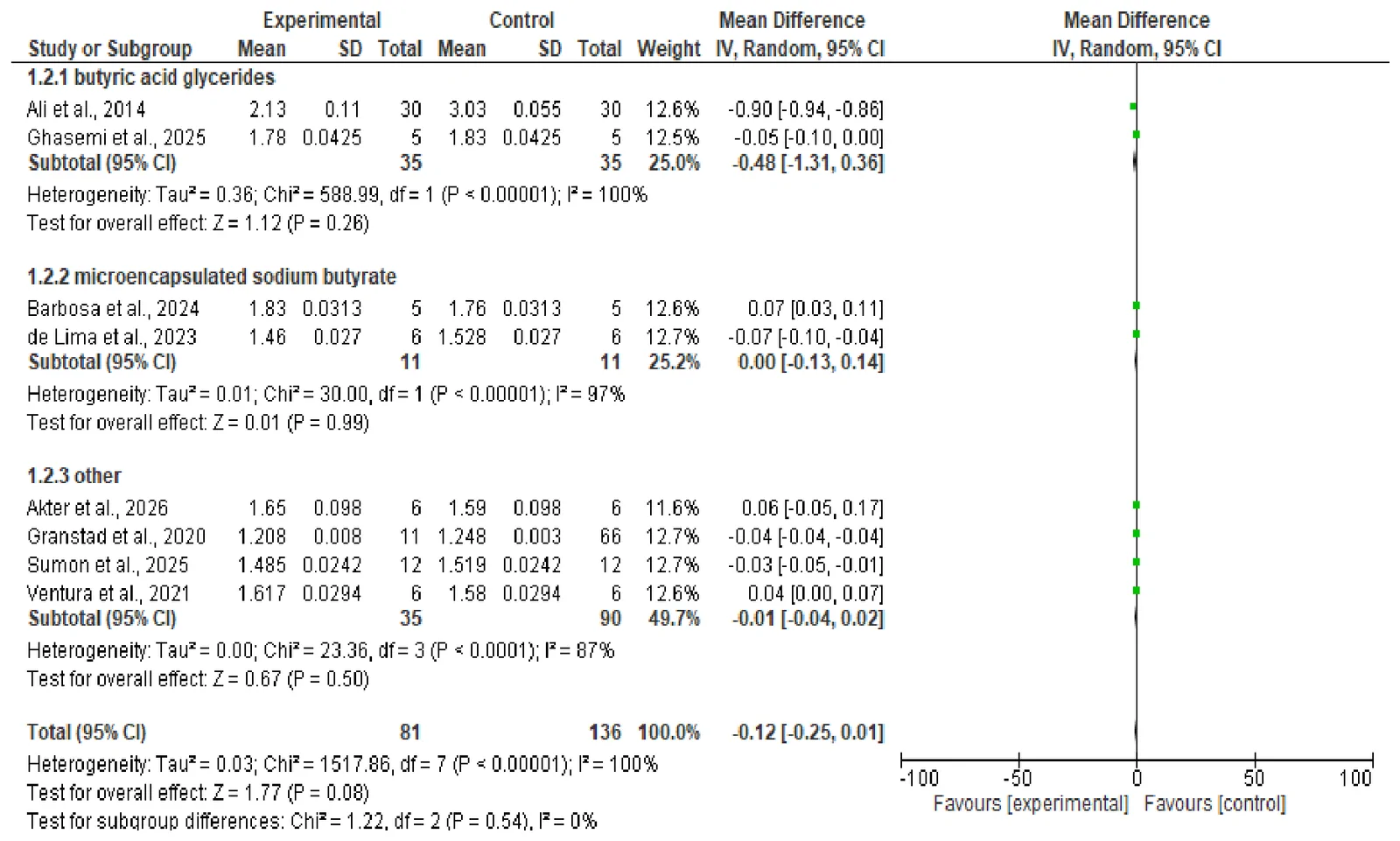
Subgroup forest plot by organic acid type for feed conversion ratio (FCR), showing no statistically significant effects across all categories and no significant subgroup differences [22,23,24,25,26,27,31,32].

Subgroup analysis based on organic acid type showed no significant effects for butyric acid glycerides (MD = −0.48 g/g, 95% CI: −1.31 to 0.36; *p* = 0.26; I^2^ = 100%), microencapsulated sodium butyrate (MD = 0.00 g/g, 95% CI: −0.13 to 0.14; *p* = 0.99; I^2^ = 97%), or other organic acids (MD = −0.01 g/g, 95% CI: −0.04 to 0.02; *p* = 0.50; I^2^ = 87%), with no significant subgroup difference (χ^2^ = 1.22, df = 2, *p* = 0.54; I^2^ = 0%), as shown in Figure 11.

**Figure 12:**
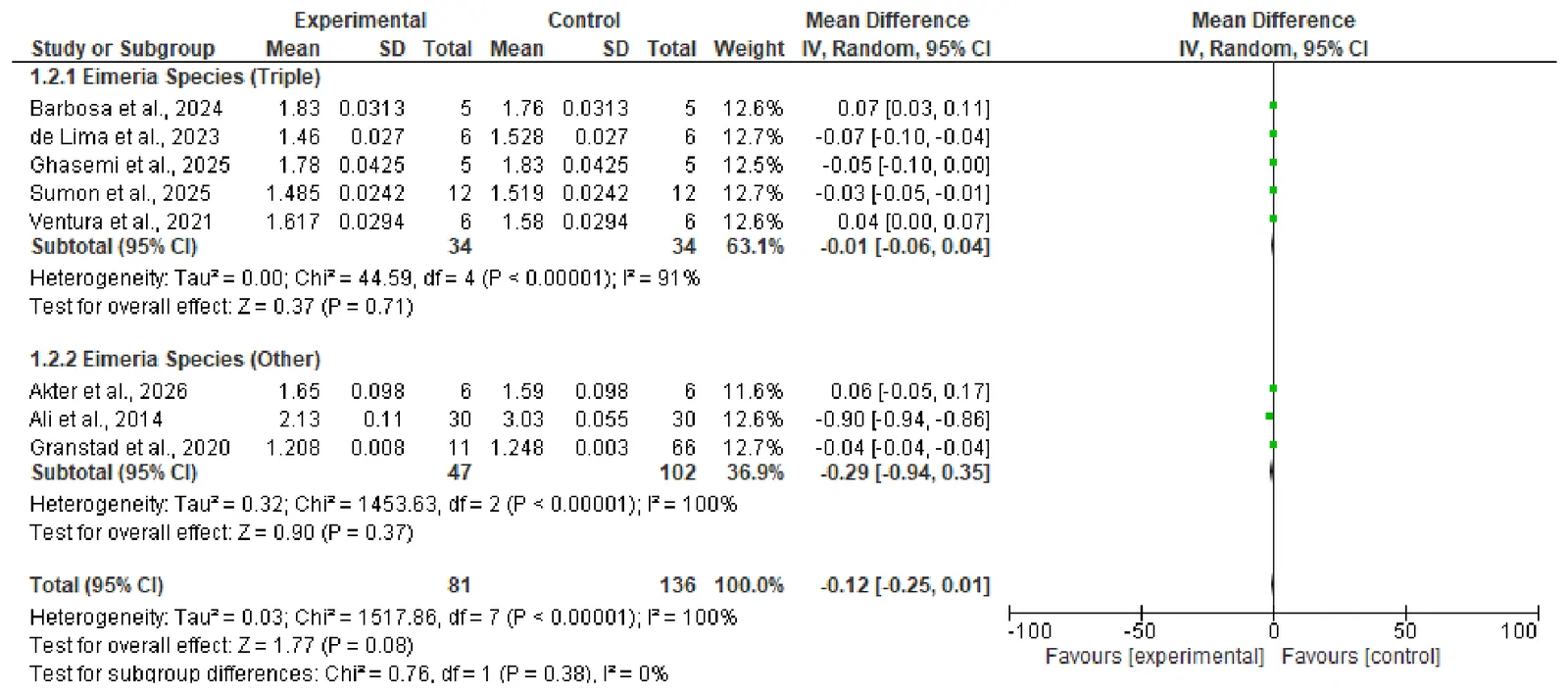
Subgroup forest plot by *Eimeria* species for feed conversion ratio (FCR), showing no statistically significant effects in any subgroup and no significant subgroup differences [22,23,24,25,26,27,31,32].

Subgroup analysis based on *Eimeria* species also showed no significant effects for triple-species infection (MD = −0.01 g/g, 95% CI: −0.06 to 0.04; *p* = 0.71; I^2^ = 91%) or other species (MD = −0.29 g/g, 95% CI: −0.94 to 0.35; *p* = 0.37; I^2^ = 100%), with no significant subgroup difference (χ^2^ = 0.76, df = 1, *p* = 0.38; I^2^ = 0%), as shown in Figure 12.

**Figure 13:**
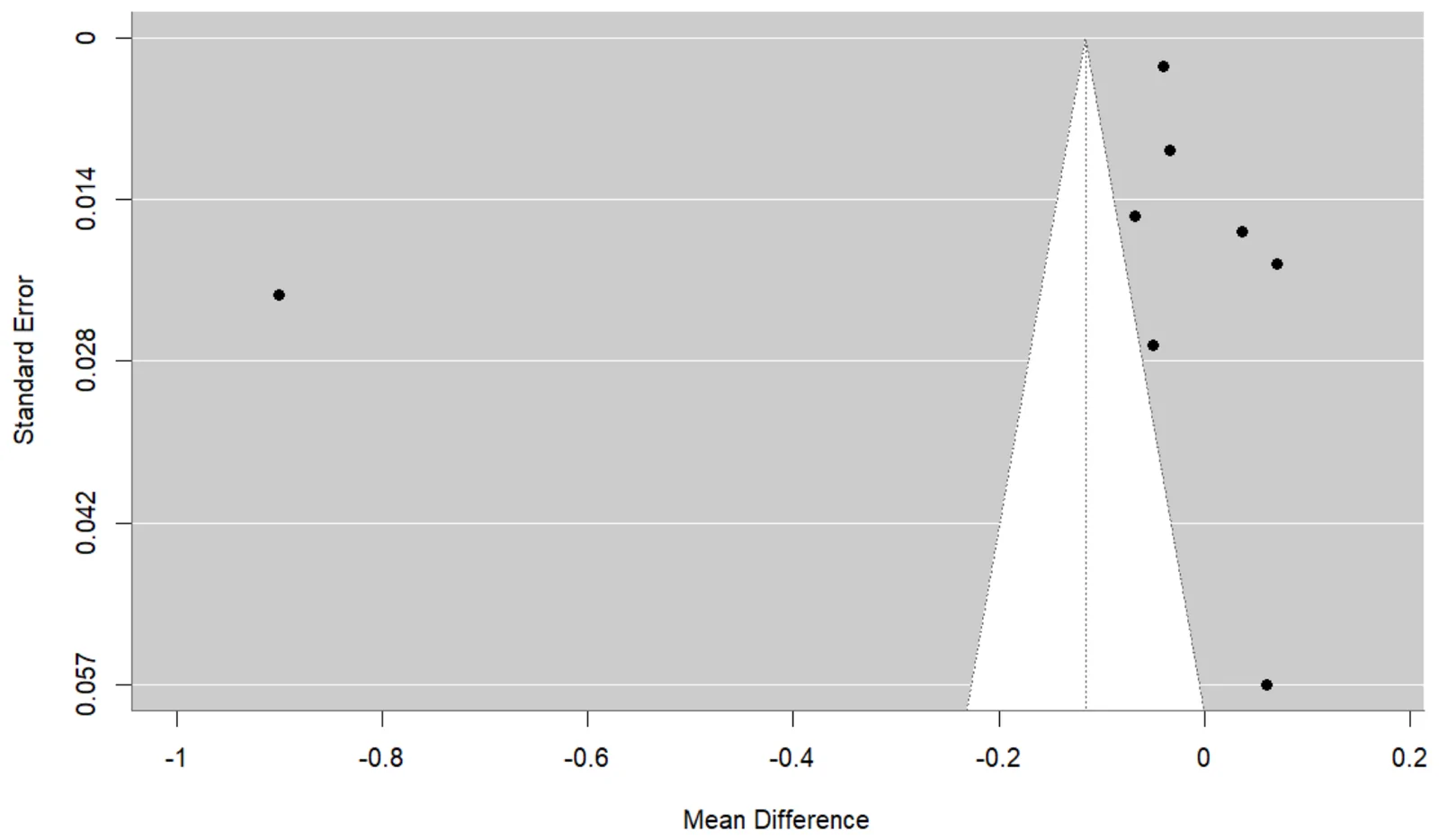
Funnel plot for feed conversion ratio (FCR).

Sensitivity analysis using the leave-one-out method showed that the pooled estimates ranged from −0.01 g/g (95% CI: −0.05 to 0.03; *p* = 0.67) to −0.14 g/g (95% CI: −0.39 to 0.11; *p* = 0.27) and remained non-significant across all iterations, indicating that no single study substantially influenced the overall results. Exploratory publication bias assessment showed no evidence of funnel plot asymmetry, which was supported by Egger’s regression test (z = 0.13, *p* = 0.89), as shown in Figure 13.

### 3.6. Lesion Score

**Figure 14:**
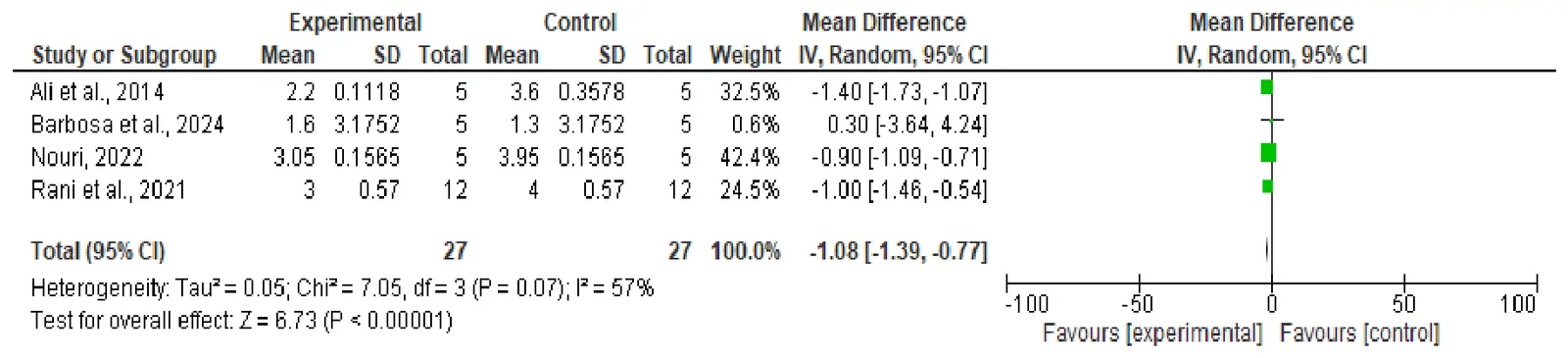
Forest plot showing the effect of organic acids on lesion score, demonstrating a statistically significant reduction with moderate heterogeneity [23,24,29,30].

The meta-analysis included 4 studies comprising 54 experimental units (27 in the treatment group and 27 in the control group). Using a random-effects model, the pooled mean difference (MD) was −1.08 score units (95% CI: −1.39 to −0.77), indicating a statistically significant effect (Z = 6.73, *p* < 0.00001). Moderate heterogeneity was observed among the included studies (τ^2^ = 0.05; χ^2^ = 7.05, df = 3, *p* = 0.07; I^2^ = 57%), as shown in Figure 14.

**Figure 15:**
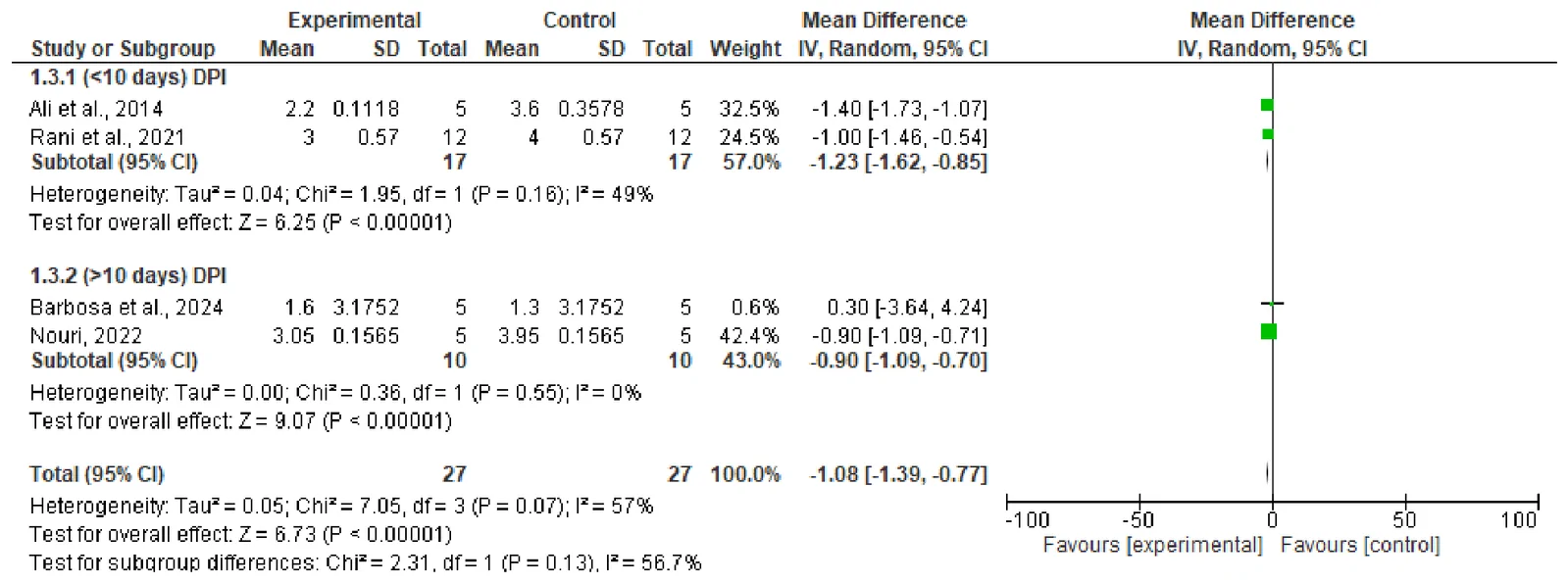
Subgroup forest plot by days post-infection (DPI) for lesion score, showing significant reductions in both <10 and >10 days groups with no significant subgroup differences [23,24,29,30].

Subgroup analysis based on days post-infection (DPI) showed significant effects for both <10 days (MD = −1.23 score units, 95% CI: −1.62 to −0.85; *p* < 0.00001; I^2^ = 49%) and >10 days (MD = −0.90 score units, 95% CI: −1.09 to −0.70; *p* < 0.00001; I^2^ = 0%), with no significant subgroup difference (χ^2^ = 2.31, df = 1, *p* = 0.13; I^2^ = 56.7%), as shown in Figure 15.

**Figure 16:**
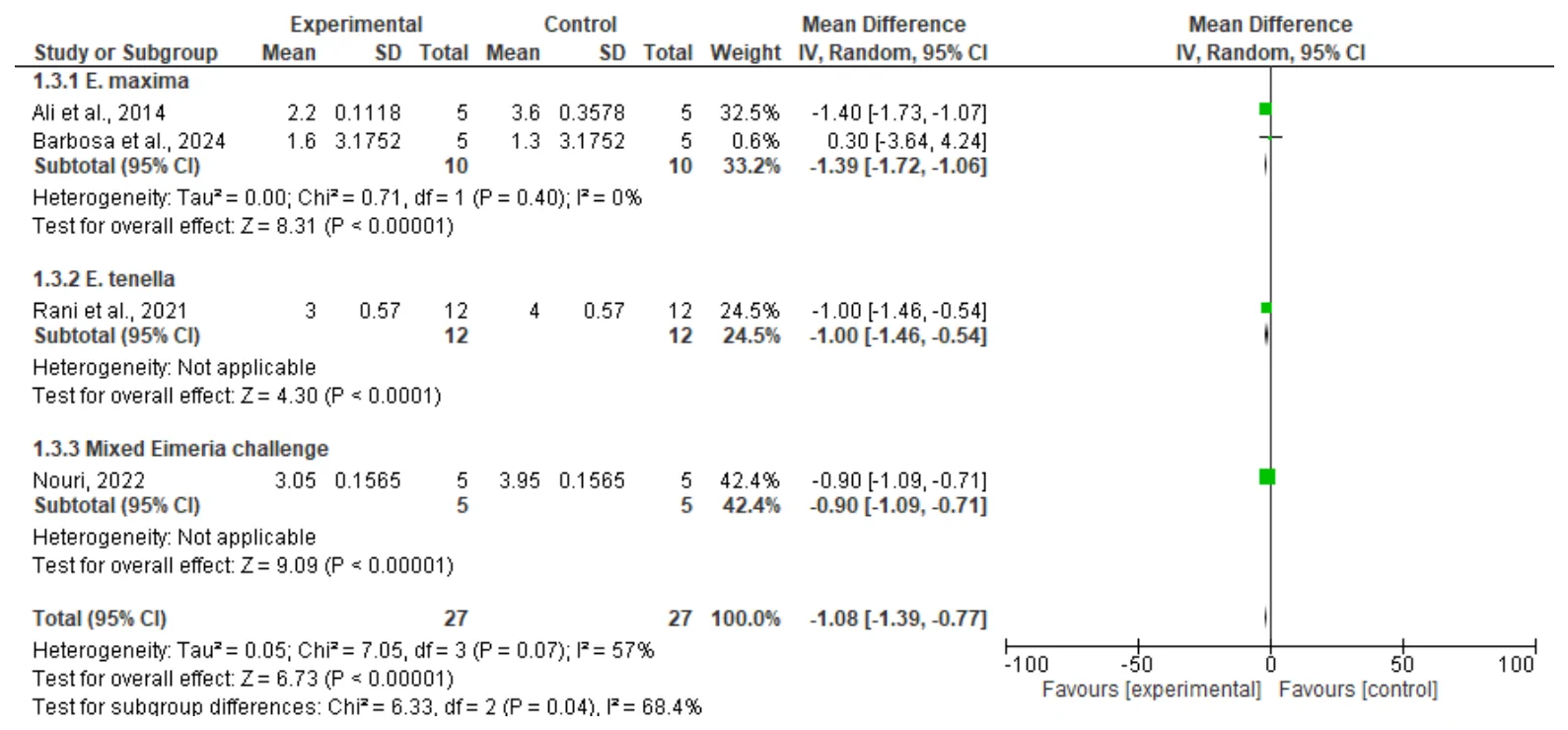
Subgroup forest plot by *Eimeria* species for lesion score, showing significant reductions across all species with a significant subgroup difference [23,24,29,30].

Subgroup analysis based on *Eimeria* species demonstrated significant effects for *E. maxima* (MD = −1.39 score units, 95% CI: −1.72 to −1.06; *p* < 0.00001; I^2^ = 0%), *E. tenella* (MD = −1.00 score units, 95% CI: −1.46 to −0.54; *p* < 0.0001), and mixed infections (MD = −0.90 score units, 95% CI: −1.09 to −0.71; *p* < 0.00001), with a significant subgroup difference (χ^2^ = 6.33, df = 2, *p* = 0.04; I^2^ = 68.4%), as shown in Figure 16.

**Figure 17:**
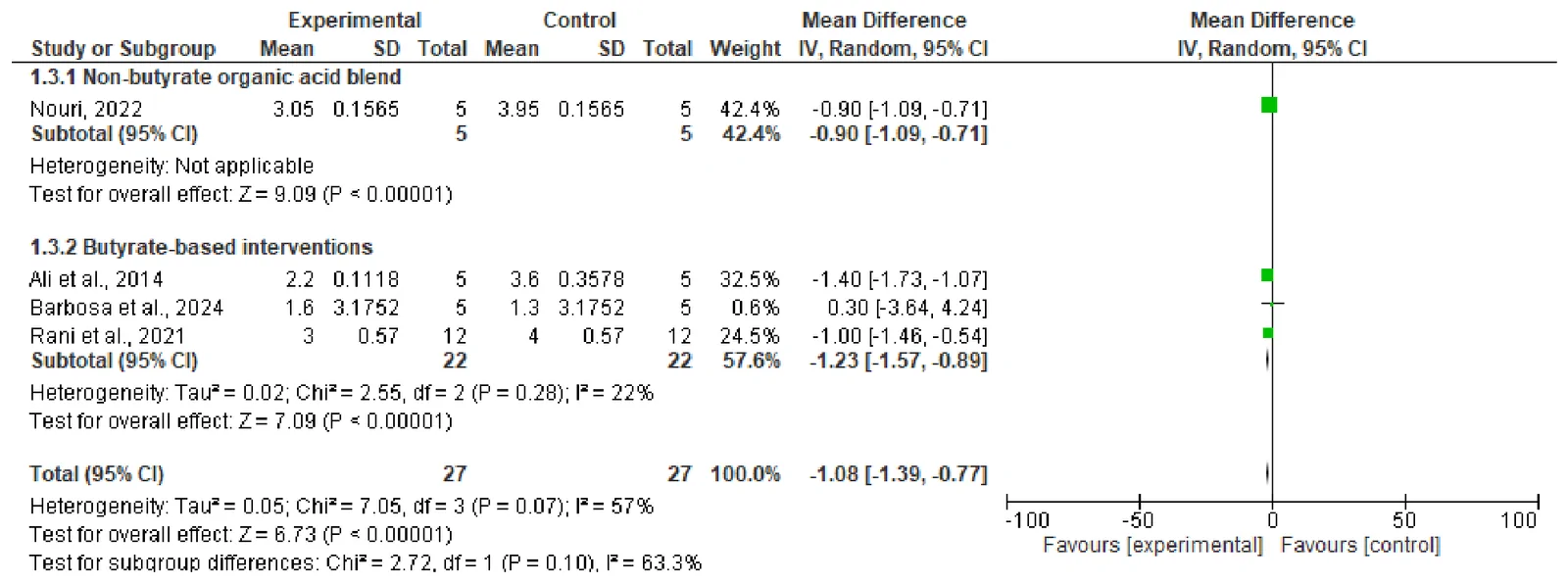
Subgroup forest plot by intervention type for lesion score, showing significant reductions for both non-butyric and butyrate-based interventions with no significant subgroup difference [23,24,29,30].

Subgroup analysis based on intervention type showed significant effects for both non-butyric organic acid blends (MD = −0.90 score units, 95% CI: −1.09 to −0.71; *p* < 0.00001) and butyrate-based interventions (MD = −1.23 score units, 95% CI: −1.57 to −0.89; *p* < 0.00001; I^2^ = 22%), with no significant subgroup difference (χ^2^ = 2.72, df = 1, *p* = 0.10; I^2^ = 63.3%), as shown in Figure 17.

**Figure 18:**
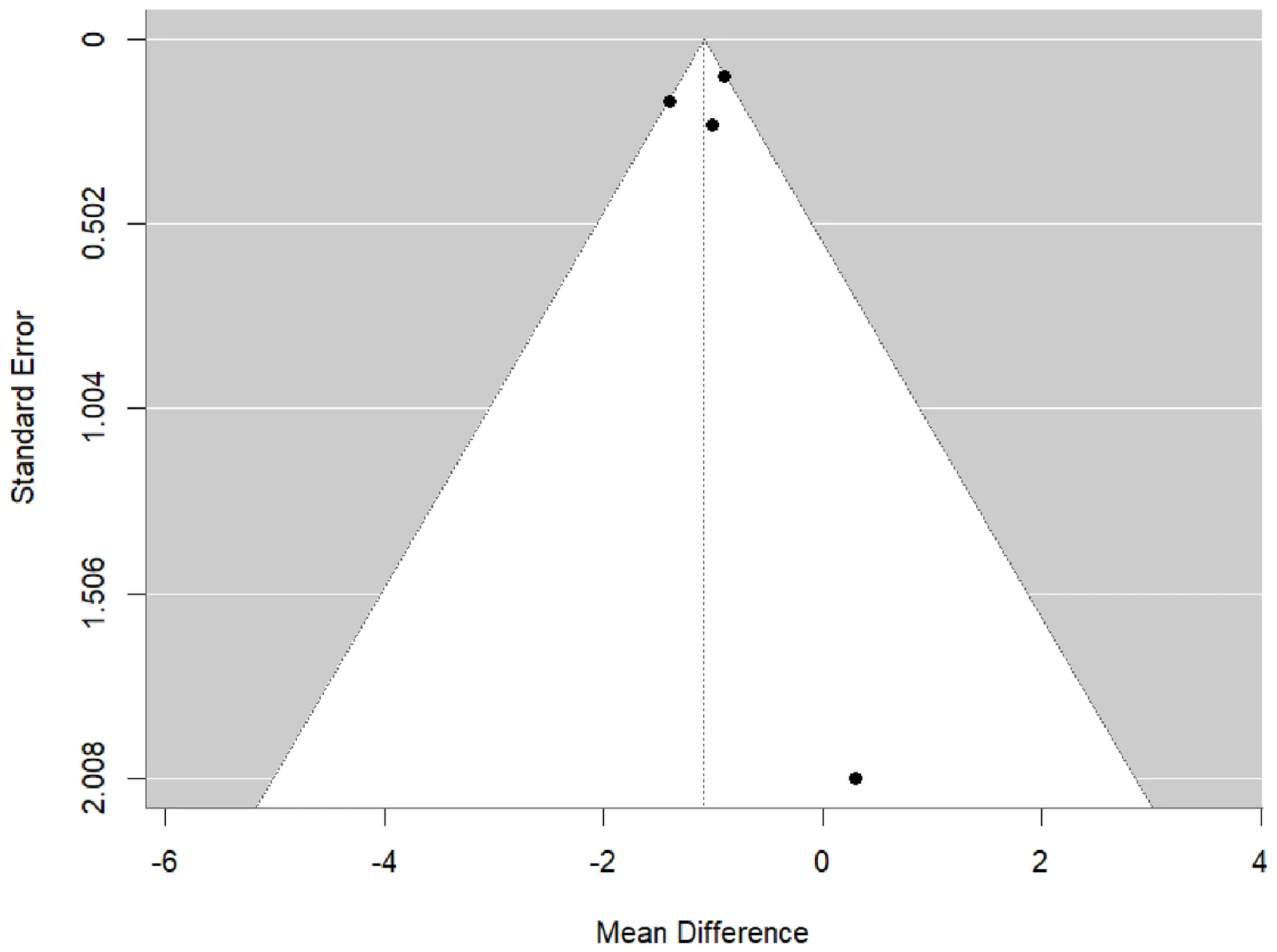
Funnel plot for lesion score.

Sensitivity analysis using the leave-one-out method showed that the pooled estimates ranged from −0.91 score units (95% CI: −1.09 to −0.73) to −1.22 score units (95% CI: −1.60 to −0.83) and remained statistically significant across all iterations, indicating that no single study substantially influenced the overall effect. Exploratory publication bias assessment showed no evidence of funnel plot asymmetry, supported by Egger’s regression test (z = 0.52, *p* = 0.60), as shown in Figure 18.

### 3.7. Oocysts per Gram (OPG)

**Figure 19:**
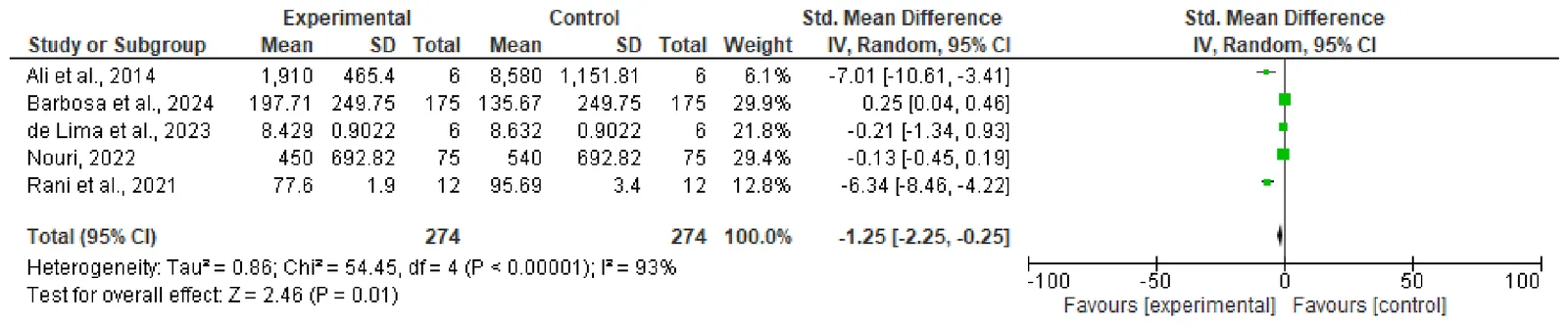
Forest plot showing the effect of organic acids on oocyst count (OPG), demonstrating a statistically significant reduction with high heterogeneity [23,24,25,29,30].

The meta-analysis included 5 studies comprising 548 experimental units (274 in the treatment group and 274 in the control group). Using a random-effects model, the pooled standardized mean difference (SMD) was −1.25 (95% CI: −2.25 to −0.25), indicating a statistically significant effect (Z = 2.46, *p* = 0.01). Substantial heterogeneity was observed among the included studies (τ^2^ = 0.86; χ^2^ = 54.45, df = 4, *p* < 0.00001; I^2^ = 93%), as shown in Figure 19.

**Figure 20:**
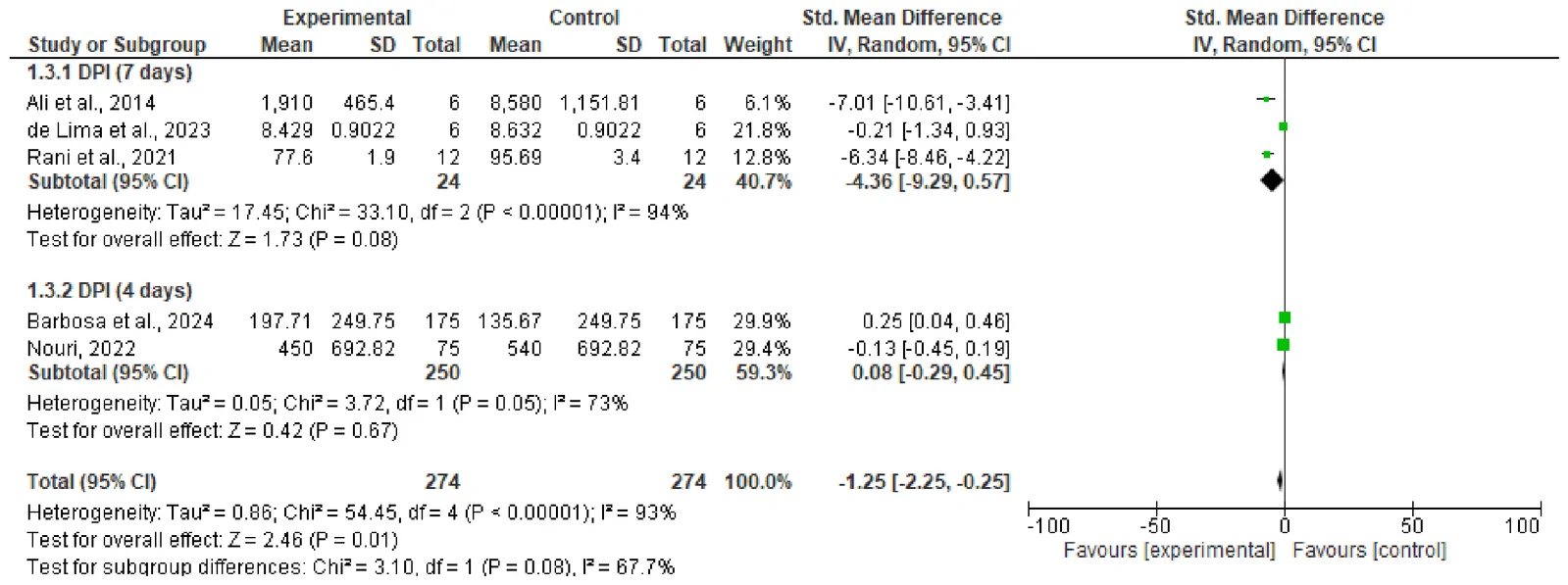
Subgroup forest plot by days post-infection (DPI) for oocyst count (OPG), showing no statistically significant effects in either 7-day or 4-day groups and no significant subgroup difference [23,24,25,29,30].

Subgroup analysis based on days post-infection (DPI) showed no statistically significant effects for studies at 7 DPI (SMD = −4.36, 95% CI: −9.29 to 0.57; *p* = 0.08; I^2^ = 94%) or 4 DPI (SMD = 0.08, 95% CI: −0.29 to 0.45; *p* = 0.67; I^2^ = 73%), with no significant subgroup difference (χ^2^ = 3.10, df = 1, *p* = 0.08; I^2^ = 67.7%), as shown in Figure 20.

**Figure 21:**
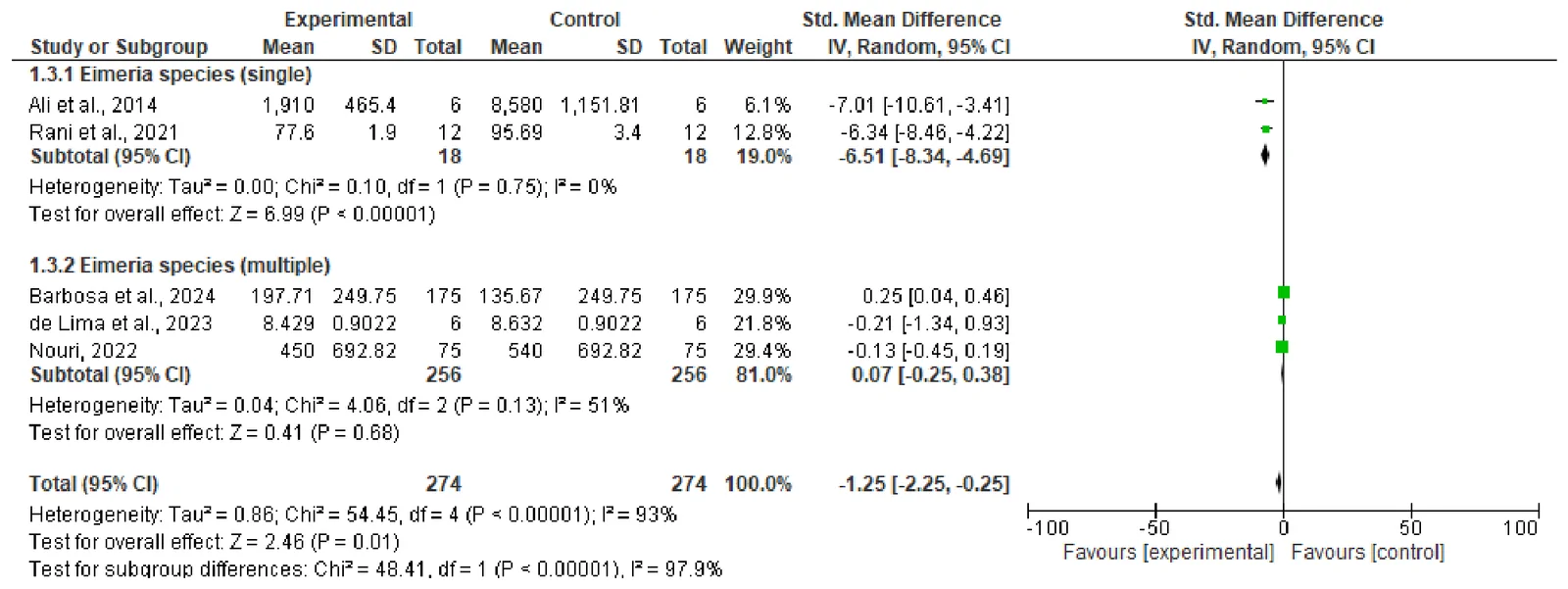
Subgroup forest plot by *Eimeria* species for oocyst count (OPG), showing a significant reduction in single-species infections but not in multiple-species infections, with a significant subgroup difference [23,24,25,29,30].

Subgroup analysis based on *Eimeria* species demonstrated a significant effect for single-species infection (SMD = −6.51, 95% CI: −8.34 to −4.69; *p* < 0.00001; I^2^ = 0%), whereas no significant effect was observed for multiple-species infection (SMD = 0.07, 95% CI: −0.25 to 0.38; *p* = 0.68; I^2^ = 51%), with a significant subgroup difference (χ^2^ = 48.41, df = 1, *p* < 0.00001; I^2^ = 97.9%), as shown in Figure 21.

**Figure 22:**
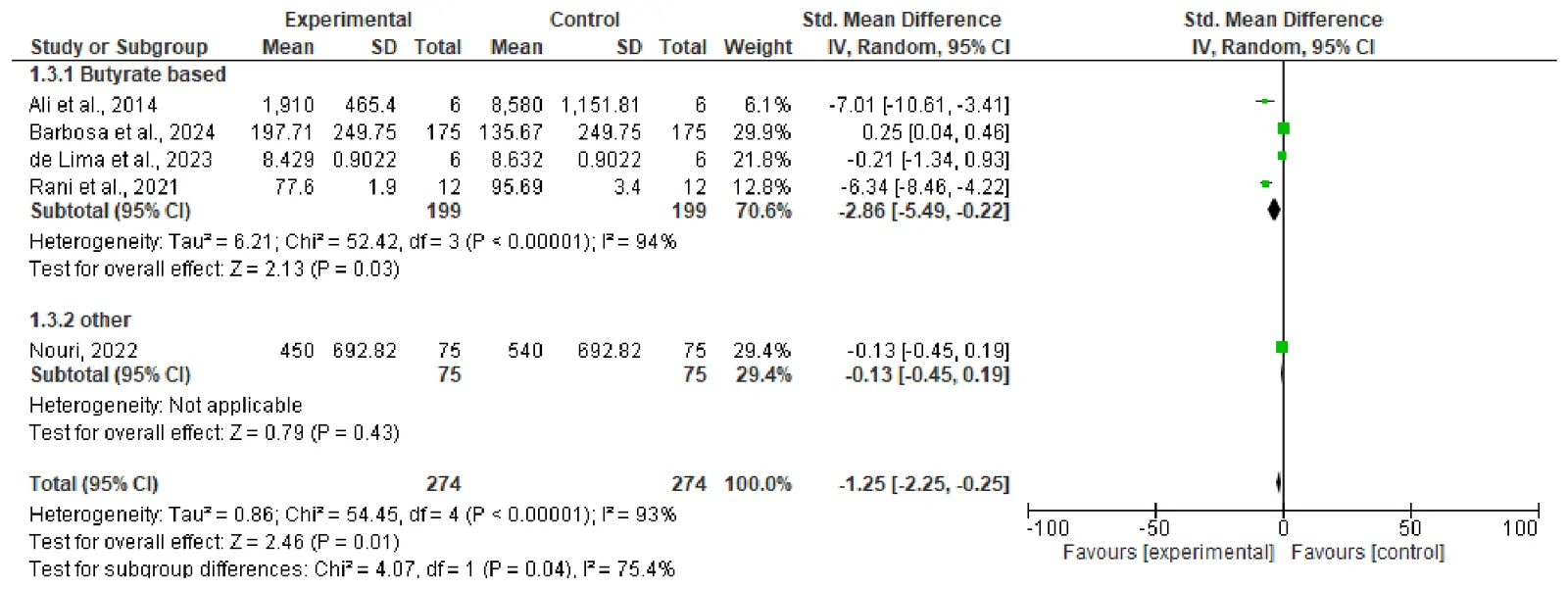
Subgroup forest plot by intervention type for oocyst count (OPG), showing a significant reduction with butyrate-based interventions but not with other organic acids, with a significant subgroup difference [23,24,25,29,30].

Subgroup analysis based on intervention type showed a significant effect for butyrate-based interventions (SMD = −2.86, 95% CI: −5.49 to −0.22; *p* = 0.03; I^2^ = 94%), while no significant effect was observed for other organic acids (SMD = −0.13, 95% CI: −0.45 to 0.19; *p* = 0.43), with a significant subgroup difference (χ^2^ = 4.07, df = 1, *p* = 0.04; I^2^ = 75.4%), as shown in Figure 22.

**Figure 23:**
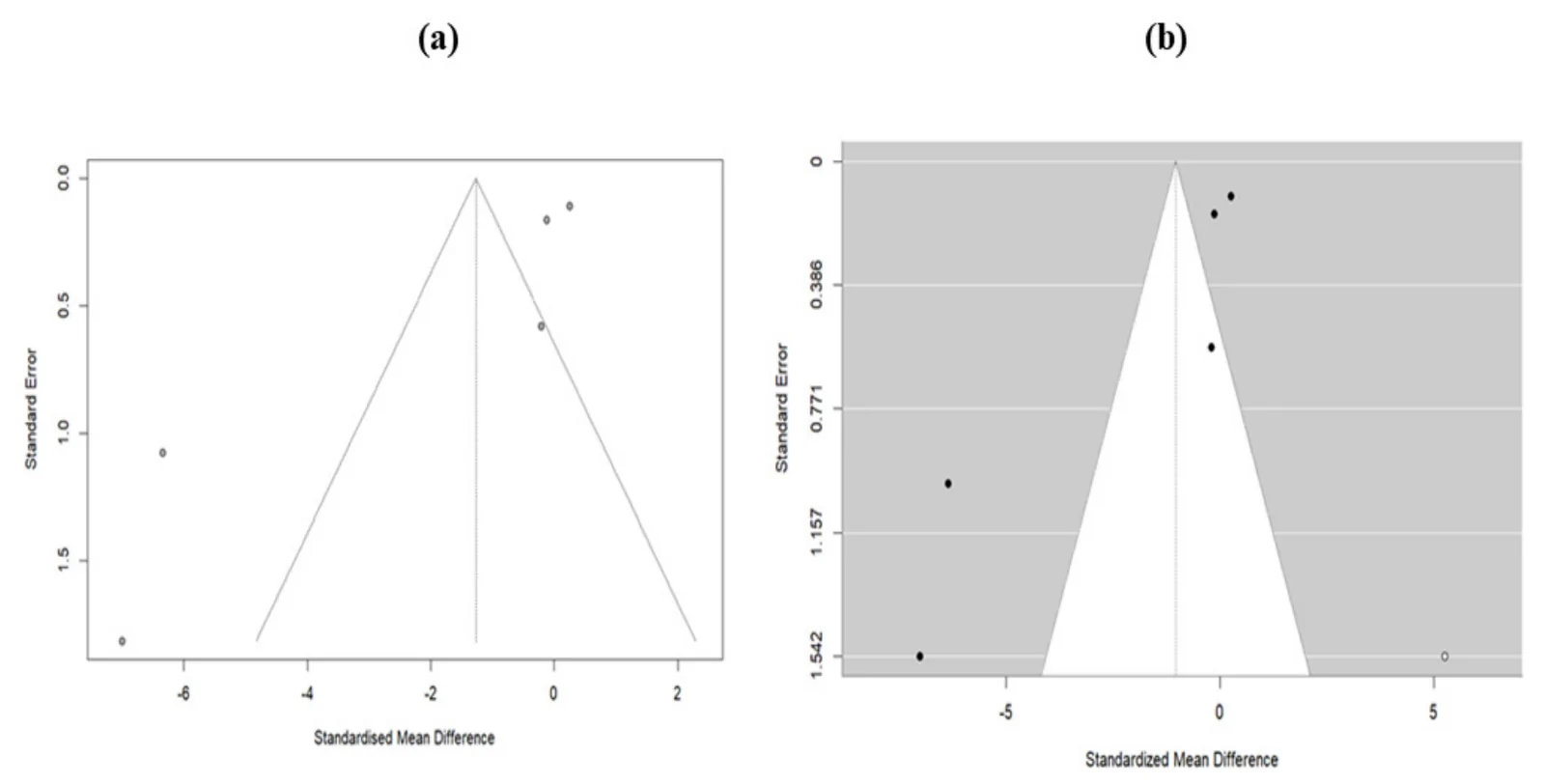
Funnel plot (**a**) and trim-and-fill analysis (**b**) for oocyst count (OPG), indicating asymmetry and potential publication bias with imputed studies adjusting the pooled estimate.

The leave-one-out sensitivity analysis demonstrated that the overall effect size remained generally consistent, with pooled estimates ranging from SMD = −0.22 (95% CI: −0.90 to 0.45; *p* = 0.52) to SMD = −2.94 (95% CI: −5.47 to −0.42; *p* = 0.02). The overall random-effects model showed a statistically significant effect (SMD = −1.27, 95% CI: −2.27 to −0.27; *p* = 0.013), and this significance was retained after omitting most individual studies, except for Ali, Seddiek [23] and Rani, Abbas [30], where the results became non-significant. High heterogeneity persisted across all iterations (I^2^ range: 84.6% to 94.5%), indicating substantial variability among studies. Exploratory publication bias assessment showed asymmetry, and Egger’s regression test indicated borderline evidence of publication bias (t = −3.10, *p* = 0.053), suggesting potential small-study effects, as shown in Figure 23.

### 3.8. Summary of Significant Subgroup Analyses

A summary of statistically significant subgroup analyses across all outcomes is presented in Table 2. Only subgroups with statistically significant effects (*p* < 0.05) are included to highlight key factors influencing the intervention outcomes.

### 3.9. Certainty of Evidence

The GRADE assessment indicated very low certainty of evidence for all four outcomes (BWG, FCR, lesion score, and OPG) as given in Table 3, primarily because of risk of bias, inconsistency, and imprecision. Indirectness was not considered serious because the included studies directly addressed the predefined population, intervention, comparator, and outcomes. Although potential small-study effects were identified for BWG and OPG, publication bias was not downgraded because the limited number of studies reduced the reliability of these assessments.

## 4. Discussion

The current meta-analysis demonstrated that organic acids showed a more consistent beneficial effect on disease-related outcomes than on growth performance in broilers challenged with coccidiosis. Pooling of the data revealed significant reductions in lesion scores and oocyst shedding; however, overall effects on body weight gain and feed conversion ratio were inconsistent. This trend is well supported by other studies reporting that organic acids reduce intestinal damage and parasite burden during *Eimeria* challenge. For example, Abdullahi, Yu [15] found a significant reduction in parasite lesion scores and oocyst indices, whereas Nouri [29] reported oocyst reduction with the use of encapsulated organic acids. Similarly, Balta, Marcu [42] and AE NASR, Sayed [54] observed reductions in lesion severity and parasite excretion, suggesting that the possible role of organic acids in controlling coccidiosis may be mainly due to their protection of the intestinal mucosa, with suppression of infection-related damage.

Mustafa, Bai [55] also demonstrated that organic acids enhanced gut health markers in challenged broilers, such as reduced lesion scores and improved intestinal morphology. In contrast, performance responses have been inconsistent across studies. Julien [17] and Mustafa, Bai [55] recently reported improvements in body weight with this treatment. However, these benefits were not consistently accompanied by improvements in both feed efficiency and parasitological outcomes. Altogether, these findings imply that organic acids could act more consistently as supportive anticoccidial and gut health-promoting agents, rather than as universal growth promoters, during coccidial challenge conditions.

The impact of organic acids on growth performance—specifically BWG and FCR—was inconsistent across studies. Although some individual studies reported positive effects on various growth parameters, these effects were not consistently observed under coccidial-challenge conditions. For instance, Julien [17] reported heavier body weight in broilers fed the organic acid/additive blend, with no significant differences in FCR. Similarly, Mustafa, Bai [55] showed improved BWG and FCR in both challenged and non-challenged birds receiving organic acids, indicating overall growth-promoting activity. In contrast, Rahmatian, Irani [56] reported that, under certain conditions, there was insufficient evidence of a clear overall improvement in performance-related parameters, although some phase-specific differences were noted.

Furthermore, Sharifuzzaman, Mun [18] reported that the beneficial effects of organic acids, especially citric acid, in improving FCR are not consistently observed, which supports the inconsistency in feed efficiency results. Supplementation strategies vary and can contribute to this inconsistency, with continuous addition of organic acids producing better growth performance than intermittent administration [57]. In contrast, Waghmare, Gupta [58] reported that flexible combinations of organic acids improved growth performance, whereas others using complex formulations or different experimental conditions showed mixed performance results. Such differences could be related to the severity of infection, dietary components, the type and dose of the organic acid used, and other physiological stresses from coccidial infection that can divert nutrients toward the immune response rather than growth.

The very high heterogeneity observed for BWG (I^2^ = 100%) indicates substantial variability in treatment effects across studies. Subgroup analyses suggested that study duration and organic acid type partly contributed to this variability, whereas *Eimeria* species did not significantly explain the between-study differences. Residual heterogeneity may reflect differences in broiler strain, challenge conditions, infection dose, supplementation level, formulation, and experimental design. Because only nine studies were available for the BWG analysis, meta-regression was not performed due to the limited statistical power and risk of unstable estimates. Therefore, the pooled BWG effect should be interpreted cautiously, as the high heterogeneity limits the precision and generalizability of the overall estimate.

The decrease in lesion scores observed in the current meta-analysis is among the most consistent and physiologically relevant effects of organic acid supplementation in coccidiosis-challenged broilers. This finding is well substantiated by several experimental studies showing the ability of organic acids to mitigate intestinal lesions caused by *Eimeria* infection. For instance, Abdullahi, Yu [15] reported a significant reduction in lesion scores in broilers supplemented with organic acids, suggesting improved intestinal integrity under challenge conditions. Similarly, Mustafa, Bai [55] reported lower jejunal lesion scores along with improvements in intestinal morphology, including increased villus height and decreased crypt depth, indicating restoration of mucosal structure.

AE NASR, Sayed [54] further confirmed that treatment with organic acids significantly decreased the severity of cecal lesions and improved histopathological parameters, like anticoccidial drugs. In addition, Balta, Marcu [42] demonstrated that mixtures of organic acids decreased tissue lesion scores in *Eimeria tenella*-challenged broilers, and this effect was associated with improved immune responses and reduced oxidative stress. The durability of these results indicates that organic acids may play diverse protective roles through various mechanisms, including stabilization of the intestinal barrier, attenuation of inflammatory pathways, and enhancement of mucosal immune responses. However, not all studies have reported significant effects; Julien [17] found no decrease in lesion scores with formulations containing organic acids, which the authors attributed to differences in other active components of these polymers or to differences in formulation complexity. In conclusion, lesion scores are a sensitive and reliable parameter for evaluating the efficacy of organic acid treatment in coccidial-induced intestinal damage.

The current meta-analysis showed a significant decrease in OPG levels, suggesting that organic acids may reduce parasite burden in coccidiosis-challenged broiler chickens; however, there was substantial heterogeneity across studies. This conclusion is consistent with multiple experimental studies demonstrating decreased oocyst shedding and parasite replication during organic acid supplementation. For instance, Abdullahi, Yu [15] reported a marked decrease in the oocyst index in the treatment group, and Nouri [29] reported higher reductions in oocyst counts with encapsulated organic acids, indicating their efficacy in controlling parasite load. Similarly, Balta, Marcu [42] showed that parasite levels (both in intestinal tissues and excreta) were reduced in pigs receiving a mixture of organic acids, which corresponded to a lower lesion severity. AE NASR, Sayed [54] also observed reduced oocyst shedding, although the effect was modest compared to that of conventional anticoccidials.

In contrast, Rahmatian, Irani [56] found that reductions in oocyst count were inconsistent at several time points, arguing that organic acids may have activity depending on the stage of infection. Furthermore, Julien [17] noted that many commonly used formulations have no significant effect on oocyst excretion. Interestingly, Park, Nam [59] suggested that decreases in oocyst shedding may be indirect, via increased host immunity and support of intestinal integrity, rather than through direct antiparasitic action. Overall, these results suggest that organic acids lower parasite burden through direct and indirect mechanisms, but this effect depends on formulation type, infection dynamics, and experimental conditions. Although the overall pooled effect on OPG was statistically significant, the DPI-specific subgroup estimates were not. This likely reflects the reduced number of studies and lower statistical power within each subgroup, together with substantial residual heterogeneity. Moreover, the test for subgroup differences was not significant (*p* = 0.08), providing insufficient evidence that the treatment effect differed between the assessed time points.

The across-outcome heterogeneity reported in this meta-analysis may be explained by methodological and biological differences among the included studies, as reflected in patterns observed in the literature. One reason for this heterogeneity is the variety of organic acid types and formulations, including single acids, blends, and encapsulated products with different modes of action and bioavailability. For example, Nouri [29] showed improved efficacy with encapsulated organic acids, indicating a potential additive effect with anticoccidials (as observed in other studies). In contrast, Julien [17] used complex formulations that included other compounds, such as essential oils and plant extracts, which may have obscured the independent effects of organic acids. The impact of supplementation strategies, such as the dose and duration, is also vital, as consistent administration has been found to produce more reproducible effects on performance than occasional use [57].

Variations due to differences in experimental conditions, such as diet composition and environmental and management practices, have been noted in broader reviews [19], which highlight the impact of these factors on study outcomes. A second major source of heterogeneity is biological variation in response to the coccidial challenge, such as differences among *Eimeria* species, infection dose, and whether infections are single or mixed [42]. In addition, the timing of outcome assessment influences effect size owing to the parasite’s dynamic lifecycle, which affects both OPG and lesion scores. These factors, combined, emphasize the complexity of interpreting pooled estimates and underscore the importance of cautious interpretation of results, as well as suggesting improvements to standardization in any future studies. Although meta-regression can quantify associations between study-level moderators and effect sizes, it was not performed because the number of studies available for individual outcomes was limited, including only nine studies for BWG and even fewer for lesion score and OPG. Evaluating multiple moderators, including *Eimeria* species, organic acid dosage, encapsulation technology, broiler age, and infection dose, would have produced an inadequate study-to-covariate ratio, low statistical power, and potentially unstable or spurious estimates. Therefore, prespecified subgroup analyses using available categorical variables were used to explore potential sources of between-study heterogeneity. These findings should be interpreted cautiously because several subgroups contained only a small number of studies. Consequently, although subgroup analyses explored organic acid type and study duration where feasible, the limited number of studies prevented formal evaluation of dose–response relationships and simultaneous adjustment for multiple moderators. Therefore, differences in dose, duration, and formulation may remain important sources of unexplained heterogeneity.

The mechanisms underlying the effects of organic acids on broilers challenged with coccidiosis involve several intertwined biological pathways related to gut integrity, immunity, and microbial modulation. Research has proven that organic acids can improve intestinal barrier function by enhancing tight junction protein expression and alleviating epithelial injury caused by *Eimeria* infection by promoting mucosal structure. For example, Mustafa, Bai [55] reported improved villus height and crypt architecture, whereas Park, Nam [59] enhanced tight junction protein production and modulated inflammatory responses. Moreover, studies have shown that organic acids decrease pro-inflammatory cytokines and modulate host immunity by increasing regional immune responses, local immunoglobulin levels, and antioxidant activity [42]. These effects contribute to decreased lesion severity and lower parasite burden.

Exploratory assessments suggested funnel-plot asymmetry for BWG and possible asymmetry for OPG. For BWG, the trim-and-fill procedure imputed potentially missing studies on the right side of the funnel plot and produced an adjusted pooled estimate of MD = 276.06 g (95% CI: 32.24 to 519.87). For OPG, the trim-and-fill analysis also suggested potentially missing studies. However, because fewer than 10 studies were available for these outcomes, funnel-plot inspection, Egger’s regression, and trim-and-fill analyses had limited statistical power and should be interpreted cautiously. Accordingly, these findings may reflect small-study effects, substantial heterogeneity, or methodological variability and should not be considered definitive evidence of publication bias.

From a commercial perspective, the practical value of organic acid supplementation appears to depend on infection pressure, formulation, and whether the additive is used alone or as part of an integrated control program. Under routine commercial conditions, organic acid supplementation may provide economic benefits; for example, Sadeghi, Janfadah [60] reported that a commercial organic acid mixture improved economic efficiency and generated returns exceeding its supplementation cost. However, that study was not conducted under a defined coccidial challenge, and its findings cannot be directly extrapolated to infected flocks. Under *Eimeria* challenge, encapsulated organic acids may offer greater practical value when used as supportive or synergistic agents. Nouri [61] showed that encapsulated organic acids improved intestinal health, oocyst reduction, immune responses, and productive performance, particularly when combined with anticoccidial drugs. In contrast, Araujo, Polycarpo [41] found that organic acids, alone or combined with probiotics, did not restore growth performance or economic returns to the level achieved with an antibiotic–anticoccidial program under a mixed *Eimeria* challenge. Collectively, these findings suggest that organic acids may be considered supportive gut-health interventions under low or subclinical infection pressure and potentially useful adjuncts under moderate challenge, especially in encapsulated formulations. Under severe coccidial pressure, however, the current evidence does not support their use as stand-alone replacements for established anticoccidial control measures. Commercial adoption should therefore consider product cost, formulation characteristics, infection pressure, and integration with vaccination, biosecurity, litter management, and veterinary anticoccidial programs. Further field trials are required to evaluate processing stability, storage stability, return on investment, and efficacy across standardized levels of coccidial challenge.

In addition, organic acids can modify the composition of the gut microbiota and favorably alter the environment, thereby inhibiting pathogen survival; these two mechanisms indirectly restrict parasite replication and oocyst excretion, as observed by Waghmare, Gupta [58] and Nouri [29]. While these strengths exist, important limitations need to be acknowledged, including high heterogeneity between studies, small sample sizes for several outcomes, differences in formulations and dosing strategies, and the inclusion of studies with combined interventions that may obfuscate the isolated effects of organic acids. In studies reporting multiple supplementation levels, the highest organic acid dose was selected for the quantitative analysis. This approach may have influenced the pooled estimates and could potentially overestimate efficacy if treatment effects increase with dose. Therefore, the pooled effects should not be interpreted as representing the average response across all supplementation levels. The literature search was restricted to the predefined databases of PubMed, Scopus, and Web of Science and did not include grey literature, postgraduate dissertations, or non-indexed conference proceedings. Consequently, the limited number of studies available for lesion score and OPG analyses may have reduced the precision and robustness of the pooled estimates. In addition, exploratory assessments suggested possible funnel-plot asymmetry and small-study effects for some outcomes. However, these analyses were underpowered because of the limited number of studies and do not permit firm conclusions regarding publication bias. However, their direction and magnitude were not uniform, and the findings should be interpreted cautiously because of the limited number of included studies. Moreover, no stratified analysis according to comparator type was performed. Therefore, potential differences in control conditions across studies may have contributed to heterogeneity and remain a limitation of the pooled estimates. Overall, these findings support the use of organic acids as complementary components of integrated coccidiosis-control programs, while further commercial-scale studies are needed to define their economic feasibility, processing stability, and effectiveness under standardized levels of infection pressure.

## 5. Conclusions

The current meta-analysis found that organic acid supplementation in coccidiosis-challenged broilers was associated with more consistent effects on disease-related outcomes than on growth performance. Very low-certainty evidence suggests that organic acids may reduce intestinal lesion severity and oocyst shedding, whereas the overall effects on body weight gain and feed conversion ratio remain uncertain. These findings indicate a potential role for organic acids as supportive gut-health and anticoccidial interventions rather than consistent growth-promoting agents under coccidial challenge. The considerable heterogeneity observed across studies may be due to variations in the type of organic acid used, the *Eimeria* species, the severity of infection, and the experimental conditions. Subgroup analyses suggested that butyrate-derived and encapsulated formulations may confer greater benefits under specific experimental conditions. However, several subgroup strata contained only a small number of studies; therefore, these subgroup findings should be regarded as exploratory and interpreted cautiously rather than as confirmatory evidence of superior efficacy. The possible mechanisms underlying the beneficial effects of organic acids may include improved intestinal transit, modulation of the immune response, and alterations in the gut microbiota, ultimately leading to increased resistance to coccidial challenge. However, the limited number of studies available for some outcomes and subgroup analyses, substantial between-study heterogeneity, and the limited power of assessments for potential small-study effects warrant cautious interpretation of the findings. Nevertheless, the available very low-certainty evidence suggests a potential role for organic acids as complementary interventions for supporting gut health and coccidiosis control in poultry, but the true magnitude and consistency of these effects remain uncertain. As such, more well-designed studies are required to fine-tune their use and determine their impact on performance responses.

## Figures and Tables

**Table 1:** Summary of Study Characteristics of Included Experimental Trials Evaluating the Effects of Organic Acid Supplementation in Broiler Chickens Challenged with Coccidiosis.

Study	Broiler Strain	Age at Challenge	*Eimeria* Species	Organic Acid	Outcomes
[22]	Ross 308 male chicks	14 days	*E. acervulina*, *E. maxima*	Sodium butyrate	BWG, FCR; Lesion score; OPG
[23]	Hubbard	14 days	*E. maxima*	Butyric acid glycerides (Lactobutyrin)	OPG; Lesion score; BWG, FCR
[24]	Male Ross^®^ broiler chicks	16 days	*E. acervulina*, *E. maxima*, *E. Tenella*	Microencapsulated sodium butyrate (MSB)	BWG, FCR; Lesion score; OPG
[25]	Ross^®^	16 days	*E. acervulina*, *E. maxima*, *E. Tenella*	Microencapsulated sodium butyrate (MSB)	FCR; Lesion score; OPG
[26]	Ross 308	14 days	*E. acervulina*, *E. maxima*, *E. Tenella*	Butyric acid glycerides (BAG)	BWG, FCR; Lesion score; OPG
[27]	Ross 308	17–18 days	*E. acervulina*, *E. mitis*, *E. tenella*, precocious *E. Maxima*	Multiple OA products (MCFA, butyrate glycerides, diformate, lactylates, OA blends)	BWG, FCR
[28]	Ross 308	17 days	NR	Butyrate product (UltraGuard™-DUO)	BWG, FCR; Lesion score
[29]	Ross 308	1 day	*E. acervulina*, *E. maxima*, *E. mitis*, *E. praecox*, *E. Tenella*	Encapsulated organic acids (lactic, acetic, benzoic, formic, citric acids)	BWG; Lesion score; OPG
[30]	Hubbard	72 days	*E. tenella*	Butyric acid	BWG, FCR; Lesion score; OPG
[31]	Cobb 500	9 days	*E. acervulina*, *E. maxima*, *E. Brunetti*	Butyric and valeric glycerides (Gastrivix™ Avi)	BWG, FCR; Lesion score; OPG
[32]	Cobb^®^ (male)	16 days	*E. acervulina*, *E. maxima*, *E. tenella*	Uncoated and microencapsulated butyric acid (alone and combined)	BWG, FCR; Lesion score

Abbreviations: BWG, body weight gain; FCR, feed conversion ratio; MCFA, medium-chain fatty acids; NR, not reported; OA, organic acid; OPG, oocysts per gram.

**Table 2:** Significant Subgroup Analyses Across Outcomes.

Outcome	Subgroup Variable	Subgroup Category	Effect Size	95% CI	*p*-Value	I^2^ (%)	Direction
BWG	Study duration	<35 days	MD = 105.62	88.39 to 122.85	<0.00001	0	Favors experimental
BWG	Organic acid type	Microencapsulated sodium butyrate	MD = −120.65	−225.67 to −15.62	0.02	75	Favors control
FCR	Study duration	<42 days	MD = −0.04	−0.05 to −0.02	<0.00001	42	Favors experimental
Lesion score	DPI	<10 days	MD = −1.23	−1.62 to −0.85	<0.00001	49	Favors experimental
Lesion score	DPI	>10 days	MD = −0.90	−1.09 to −0.70	<0.00001	0	Favors experimental
Lesion score	*Eimeria* species	*E. maxima*	MD = −1.39	−1.72 to −1.06	<0.00001	0	Favors experimental
Lesion score	*Eimeria* species	*E. tenella*	MD = −1.00	−1.46 to −0.54	<0.0001	NR	Favors experimental
Lesion score	*Eimeria* species	Mixed infection	MD = −0.90	−1.09 to −0.71	<0.00001	NR	Favors experimental
Lesion score	Intervention type	Non-butyric organic acid blend	MD = −0.90	−1.09 to −0.71	<0.00001	NR	Favors experimental
Lesion score	Intervention type	Butyrate-based interventions	MD = −1.23	−1.57 to −0.89	<0.00001	22	Favors experimental
OPG	*Eimeria* species	Single-species infection	SMD = −6.51	−8.34 to −4.69	<0.00001	0	Favors experimental
OPG	Intervention type	Butyrate-based interventions	SMD = −2.86	−5.49 to −0.22	0.03	94	Favors experimental

BWG, body weight gain; FCR, feed conversion ratio; OPG, oocysts per gram; MD, mean difference; SMD, standardized mean difference; CI, confidence interval; DPI, days post-infection.

**Table 3:** GRADE Assessment of the Certainty of Evidence for the Primary Outcomes.

Outcome	Studies/Units	Effect Estimate	Risk of Bias	Inconsistency	Indirectness	Imprecision	Publication Bias	Certainty
BWG	9/233	MD 49.88 g (95% CI −156.94 to 256.70)	Serious (−1)	Very serious (−2)	Not serious	Serious (−1)	Suspected; no downgrade	Very low
FCR	8/217	MD −0.12 g/g (95% CI −0.25 to 0.01)	Serious (−1)	Very serious (−2)	Not serious	Serious (−1)	No downgrade	Very low
Lesion score	4/54	MD −1.08 score units (95% CI −1.39 to −0.77)	Serious (−1)	Serious (−1)	Not serious	Serious (−1)	No downgrade	Very low
OPG	5/548	SMD −1.25 (95% CI −2.25 to −0.25)	Serious (−1)	Very serious (−2)	Not serious	Serious (−1)	Suspected; no downgrade	Very low

GRADE certainty categories: high, moderate, low, and very low. Downgrading by −1 indicates serious concern and −2 indicates very serious concern. BWG, body weight gain; FCR, feed conversion ratio; OPG, oocysts per gram; MD, mean difference; SMD, standardized mean difference; CI, confidence interval.

## Data Availability

The original contributions presented in this study are included in the article. Further inquiries can be directed to the corresponding authors.

## Supplementary Materials

The following supporting information can be downloaded at: https://www.mdpi.com/article/10.3390/vetsci13090941/s1, Table S1: Database-specific search strategies used for the systematic literature search; Table S2: Characteristics of the studies included in the systematic review and meta-analysis.
