# Supplementary material for: Effects of Dietary Organic Acid Supplementation in Coccidiosis-Challenged Broilers: A Meta-Analysis
Source: Vet Sci. 2026 Sep 10;13(9):941. doi: 10.3390/vetsci13090941 (PMC13612061; doi:10.3390/vetsci13090941)
Supplement: Supplementary file 1 [file vetsci-13-00941-s001.zip › vetsci-4473280-supplementary.pdf]

Table S1: Database-specific search strategies used for the systematic literature search

Database	Query
**PubMed**	((((“Chickens”[MeSH] OR broiler*[tiab] OR “broiler chicken*”[tiab] OR poultry[tiab])) AND ((“Coccidiosis”[MeSH] OR “Eimeria”[MeSH] OR coccidiosis[tiab] OR eimeria[tiab] OR “Eimeria spp”[tiab]))) AND ((“Organic Acids”[MeSH] OR “Fatty Acids, Volatile”[MeSH] OR “Butyric Acid”[MeSH] OR “Formic Acid”[MeSH] OR “Acetic Acid”[MeSH] OR “Propionic Acid”[MeSH] OR “Citric Acid”[MeSH] OR “Lactic Acid”[MeSH] OR “Fumaric Acid”[MeSH] OR “Organic acid*”[tiab] OR “acidifier*”[tiab] OR “short chain fatty acid*”[tiab] OR SCFA[tiab] OR butyrate[tiab] OR propionate[tiab] OR formate[tiab] OR acetate[tiab] OR “acid blend*”[tiab]))) AND ((“Feed Conversion Ratio”[tiab] OR FCR[tiab] OR “Body Weight Gain”[tiab] OR BWG[tiab] OR “weight gain”[tiab] OR “lesion score*”[tiab] OR “oocyst count*”[tiab] OR OPG[tiab]))
**Scopus**	TITLE-ABS-KEY ((broiler* OR “broiler chicken*” OR poultry) AND (coccidiosis OR eimeria OR “Eimeria spp”) AND (“organic acid*” OR acidifier* OR “short chain fatty acid*” OR SCFA OR butyrate OR propionate OR formate OR acetate OR “acid blend*” OR “dietary acid*”) AND (“feed conversion ratio” OR FCR OR “body weight gain” OR BWG OR “weight gain” OR “lesion score*” OR “oocyst count*” OR OPG))
**WOS**	TS=((broiler* OR “broiler chicken*” OR poultry) AND (coccidiosis OR eimeria OR “Eimeria spp”) AND (“organic acid*” OR acidifier* OR “short chain fatty acid*” OR SCFA OR butyrate OR propionate OR formate OR acetate OR “acid blend*” OR “dietary acid*”) AND (“feed conversion ratio” OR FCR OR “body weight gain” OR BWG OR “weight gain” OR “lesion score*” OR “oocyst count*” OR OPG))

Table S2: Characteristics of the Studies Included in The Systematic Review and Meta-Analysis.

Author, Year	Country	Total Sample Size	Broiler Strain	Age At Challenge	Eimeria Species	Infection Dose	Day Of Outcome Measurement	Organic Acid Type	Dose And Duration	Comparator	Outcomes Reported
(Akter et al., 2026)	Australia	576	Ross 308 male chicks	14 days	E. acervulina and E. maxima	1 mL containing 5,000 oocysts each of E. acervulina and E. maxima	BWG and FCR: days 10, 21, and 35; Lesion score: day 21; OPG: days 17–19, 20–22, 23–25, and 26–28	Sodium butyrate	0.08% sodium butyrate; fed from starter to finisher phase (days 0–35)	Positive control diet: Standard wheat-soybean meal-based diet without additives, challenged with Eimeria spp.	BWG, FCR, Lesion score, OPG
(Ali et al., 2014)	Egypt	180	Hubbard	14 d of age	E. maxima	1 × 10^4 sporulated oocysts	OPG: 5–14 d PI; Lesion score: 8th d PI; BWG/FCR: d 42	butyric acid glycerides (Lactobutyrin)	4 g/kg ration; duration: NR	infected and non-treated	Feed Conversion Ratio (FCR); Body Weight Gain (BWG); Lesion score; Oocyst count (OPG)
(Barbosa et al., 2024)	NR	1050	male Ross® broiler chicks	16 d.o.	Eimeria acervulina, E. maxima and E. tenella	0.5 mL suspension containing 2×10^5 sporulated oocysts/broiler of E. acervulina and 2×10^4 sporulated oocysts/broiler of E. maxima and E. tenella	FCR/BWG: 21 and 42 d.o.; OPG: 4, 5, 6, and 7 days after inoculation; Lesion score: 21 and 42 d.o.	microencapsulated sodium butyrate (MSB)	1,000, 1,500, or 2,000 mg/kg; 1 to 42 d.o.	CC - challenged control diet	Feed Conversion Ratio (FCR); Body Weight Gain (BWG); Lesion score; Oocyst count (OPG)
(de Lima et al., 2023)	Brazil	1200	Ross®	16 d-old	*Eimeria acervulina*, *E. maxima*, and *E. tenella*	2 × 10^5 sporulated oocysts/bird of *E. acervulina* and 2 × 10^4 for *E. maxima* and *E. tenella*	FCR: 21 and 42 d-old; OPG: 4, 5, 6, and 7 days post-inoculation; Lesion score: 5, 6, 7, 8, 10, 12, and 19 days post-inoculation	microencapsulated sodium butyrate (MSB)	1,000 mg/kg, 1,500 mg/kg, or 2,000 mg/kg; 1–42 d-old	negative control (NC) - basal diet (BD), with no antibiotic; positive control (PC) - BD + antibiotic avilamycin	Feed Conversion Ratio (FCR); Lesion score; Oocyst count (OPG)
(Ghasemi et al., 2025)	Iran	550	Ross 308	day 14	*Eimeria acervulina*, *Eimeria maxima*,	1 mL of a solution containing approximatel	days 1, 10, 24, and 42; day 21 (7 days after oocyst inoculation); 6–8, 8–	butyric acid glycerides (BAG)	2 g/kg BAG; duration NR	positive control group (PC; coccidiosis-challenged + basal	FCR, BWG, Lesion score, OPG

					and *Eimeria tenella*	y 50,000, 10,000, and 5,000 oocysts of *Eimeria acervulina*, *Eimeria maxima*, and *Eimeria tenella*, respectively	10, and 10–12 days post-infection			diet without supplementation)	
(Granstad et al., 2020)	Norway	5280	Ross 308	day 17 (one trial) or 18 (five trials) post hatch	*Eimeria acervulina*, *Eimeria mitis*, *Eimeria tenella*, and two precocious lines of *Eimeria maxima*	10-fold dose of Paracox-5 vet. containing live, sporulated oocysts from five attenuated strains of *Eimeria* spp. in the drinking water: *E. acervulina* (approximately 5750 oocysts per broiler), *E. mitis* (approximately 11,500 oocysts), *E. tenella* (approximately 5750 oocysts), and two precocious lines of *E. maxima* (approximately 3450 oocysts)	days 0, 14 and 28; BWG and FCR calculated for days 0–14, 14–28 and 0–28	Medium-chain fatty acids (C6, C8 and C10); Tri- and diglycerides of butyric acid (C4); Diformate derived from C1 (57% Na-formate, 39% formic acid); Lactylates (C12 and C14 esterified with lactic acid); OA-containing blends: benzoic acid (80%–83%) and a blend of essential oils; short- and medium chain fatty acids (including C4 and C12), phenolic compound and organic acids; short- and medium-chain fatty acids (including monoglycerides of C3, C4, C8 and C10) and essential oils; glycerol-esterified short- and medium-chain fatty acids (including C3, C4, C8 and C10) and 6% phytogenics	Inclusion rate recommended by the manufacturers; OA products dosages (starter/grower, g/ton feed): 1600/1600, 1000/1000, 3000/3000, 750/750; OA-containing blends: 300/300, 1500/1500, 3000/2500, 750/750; from 0 to 28 days of age	Negative control group (NEG) receiving feed with neither antimicrobial feed additives (AGPs or coccidiostats) nor ATA products	Feed Conversion Ratio (FCR); Body Weight Gain (BWG)
(Naghizadeh et al., 2022)	NR	672	Ross 308	day 17	NR	10 times overdose of an attenuated live vaccine against coccidiosis	day 21, 23, 24, 29, and 34; 11–23, 24–34, and 1–34	butyrate product UltraGuard™-DUO	0.5 g/kg feed; from day one and onwards	no dietary supplement (control)	Feed Conversion Ratio (FCR), Body Weight Gain (BWG), Lesion score
(Nouri, 2022)	Iran	525	Ross 308	1 day of age	*Eimeria acervulina*, *Eimeria maxima*, *Eimeria mitis*, *Eimeria praecox*, and *Eimeria tenella*	Eimeria oocysts in the used litter for all cited treatment groups was ~10^7/g (± 1.80 SD)	BWG: 22 days of age; Lesion score: 22 days of age [21 DPI]; OPG: 4, 7, 10, 13, 19, 22, 25, 28, 31, 34, 37, 42 days of the experimental period	Encapsulated organic acids (EOAs; comprised of 15% lactic acid, 10% acetic acid, 5% benzoic acid, 5% formic acid, and 5% citric acid)	0.1% EOAs; from hatch in diet	Positive control group (T2): used litter from a poultry farm challenged with *Eimeria* spp.; diet without additives	BWG, Lesion score, Oocyst count (OPG)

(Rani et al., 2021)	Pakistan	72	Hubbard	72 days old	*Eimeria tenella*	50,000 sporulated oocysts	Weekly until the completion of the experiment; 7th day post inoculation; 7th day post infection; 14th day post infection	Butyric acid	1.2%, 1.0%, and 0.8% through diet; duration NR	Group D (positive control) was maintained as infective and treated control; Group E (negative control) was maintained as infective and non-treated control; Group F (control) was maintained as non-infective and non-treated control	FCR; BWG; Lesion score; Oocyst count (OPG)
(Sumon et al., 2025)	Australia	960	Cobb 500	d9	*E. acervulina*, *E. maxima* and *E. brunetti*	one mL of live sporulated *Eimeria* strains containing *E. acervulina* (5000 oocysts), *E. maxima* (5000 oocysts) and *E. brunetti* (2500 oocysts)	FCR and BWG: d8, d19, d35, and overall d0-35; Lesion score: d16; Oocyst count (OPG): d16 and d20	blend of butyric and valeric glycerides (Gastrivix™ Avi)	500 g/ton (starter, d0-8), 500 g/ton (grower, d8-19), and 250 g/ton (finisher, d19-35)	CC: challenged control	Feed Conversion Ratio (FCR); Body Weight Gain (BWG); Lesion score; Oocyst count (OPG)
(Ventura et al., 2021)	Brazil	1320	one-day-old male Cobb® chicks	16 days of age	*Eimeria acervulina*, *E. maxima*, and *E. tenella*	0.5 ml of suspension containing 2 × 10^5 sporulated oocysts/birds of *E. acervulina* and 2 × 10^4 *E. maxima* and *E. tenella*	BWG and FCR: 1–21 d and 1–42 d; Lesion score: 21, 22, 23, 24, 25, 26, 28 and 35 d of age	uncoated butyric acid; microencapsulated butyric acid; uncoated butyric acid + microencapsulated butyric acid	Initial phase (1–21 d): 1 kg/t of M and U treatments; Final phase (22–42 d): 0.5 kg/t. Starter: isolated butyric acids, 0.1% butyric acid; associated acids, 0.05% uncoated butyric acid + 0.05% microencapsulated acid. Grower: isolated butyric acids, 0.05% butyric acid; associated acids, 0.025% uncoated butyric acid + 0.025% microencapsulated acid	negative control (NC) – basal diet (BD) with no anticoccidial or antibiotic; positive control (PC) – BD + antibiotic and anticoccidial	BWG; FCR; Lesion score
